# Mask Wearing as Cultural Behavior: An Investigation Across 45 U.S. States During the COVID-19 Pandemic

**DOI:** 10.3389/fpsyg.2021.648692

**Published:** 2021-07-21

**Authors:** Markus Kemmelmeier, Waleed A. Jami

**Affiliations:** Interdisciplinary Social Psychology Ph.D. Program, University of Nevada, Reno, NV, United States

**Keywords:** facial coverings, COVID-19, independence, interdependence, tightness-looseness, honor culture, conservatism

## Abstract

Although masks (face coverings) are a prime tool in fighting airborne pathogens, during the COVID-19 pandemic in the United States the use of masks encountered resistance based on existing patterns of cultural division. We argue that mask wearing must be understood basis on existing cultural frames assessed at both the individual level and the state level. We relied on prominent frameworks in cultural psychology: individualism-collectivism as well as independent and interdependent self-construals, the tightness-looseness framework, U.S. honor cultures, and political orientation as predictors. Using multilevel modeling, in a sample of 633 respondents from 45 U.S. states we investigated mask-wearing behavior, masks' perceived utility, implications for well-being, and the social meaning attributed to masks. Conservatism was linked to lower mask wearing, and consistently unfavorable perceptions of mask wearing. Collective interdependence predicted favorable perceptions of masks, as did state-level differences in collectivism; both constructs were linked with viewing mask wearing to be normative. Independent self-construal predicted a greater intent to wear masks, even though masks were also evaluated less favorably. Mediation analyses revealed that a single mediator, the perceived utility of mask wearing, was implicated in translating the effects of different cultural predictors into behavior. Additional findings highlighted that in tightener (vs. looser) states masks wearing was conceived of as a civic duty, whereas in U.S. honor states mask were seen as spoiling one's public image. Our discussion focuses on the cultural and political context of mask wearing, argues that different communities in the U.S. respond to its symbolic and social meaning, and suggest strategies to increase mask wearing among those who are otherwise reluctant to do so.

## Introduction

Facial coverings, conventionally referred to as “masks,” are a prime tool in fighting airborne pathogens (e.g., Davies et al., 2013; Konda et al., 2020; Leung et al., 2020; Roberge and Roberge, 2020; Perra, 2021). Though surgical facemasks and N95 respirators are more effective in inhibiting transmission, cloth face coverings do offer some level of protection. Wearing a face mask was common practice in East Asian countries during the outbreak of SARS in 2002, and various flu epidemics (e.g., Wu et al., 2004). However, as the United States has struggled with the COVID-19 pandemic, face masks have turned into a public symbol of division between different segments of the U.S. population. At the time when the present research was conducted (late July 2020), the United States had over 4 million documented cases, by far the country with the highest number of infections in the world. Yet, there has been considerable resistance against wearing masks (e.g., Haischer et al., 2020). Controversies erupted that were based on the symbolic meaning of masks, not necessarily their effectiveness as a tool in reducing infection (cf. Timpka and Nyce, 2021). More generally, COVID-19 has revealed pronounced cultural differences that may underpin how different populations respond to epidemics [see Van Bavel et al. (2020)]. In this paper, we argue that masks and the controversies over mask wearing are mapped onto existing patterns of cultural division and political polarization. Specifically, we argue that established frameworks in cultural and political psychology help elucidate the controversies over mask wearing.

### Masks in the U.S. During 2020 COVID-19

The first documented cases of COVID-19 in the U.S. emerged during January 2020, with the first documented deaths occurring at the end of that month. On January 30, 2020, the WHO Director General declared COVID-19 to be a “Public Health Emergency of International Concern” and on January 31, 2020, the Center for Disease Control and Prevention (CDC) followed suit by declaring the coronavirus a public health emergency. Although cases mounted slowly, toward the beginning of the U.S. epidemic, public health authorities did not recommend wearing masks. As late as March 2020, infectious disease expert Dr. Anthony Fauci and other public officials did not encourage Americans to wear masks. This stance was driven in part by the concern that the supply of medical masks and other personal protective equipment (PPE) was insufficient, and public officials did not wish for the general public to compete with medical professionals and health workers over limited quantities. Moreover, there was some uncertainty over the effectiveness of masks. Whereas N95 respirator masks were specifically designed to prevent airborne infections, their supplies, as that of (surgical) procedure masks, were quickly exhausted or reserved for health care institutions. This left the general public only to use regular cloth masks, whose ability to prevent infection was inferior relative to N95 and procedure masks (e.g., Asadi et al., 2020; Whiley et al., 2020). However, officials quickly emphasized that regular cloth masks were at least moderately effective at reducing the spread of the coronavirus, if worn by infected individuals. This was critical, as many carriers of the virus were asymptomatic, and individuals with the virus were particularly contagious during the days immediately prior to the initial manifestation of symptoms (Li et al., 2020a). In other words, the ability of cloth masks to protect the wearer from infection remained limited and was not necessarily perceived to be in the immediate self-interest of the person. Rather, wearing a cloth mask was effectively a behavior that protected others and members of the wider community—a collective behavior from which individuals primarily benefitted by limiting overall community spread. Recognizing the urgent need to move to widespread public health measures to reign in an escalating pandemic, on April 2, 2020, the CDC began advising Americans to wear masks in public.

Whereas U.S. federal institutions do not have any authority to impose far-reaching public health restrictions, many U.S. states and cities eventually passed executive orders and ordinances, resulting in temporary shutdowns, curfews, and limits on public gatherings. In an attempt to limit community spread, numerous jurisdictions also issued mandates that made wearing masks compulsory in public places. Such rules were often met with protests, including ones involving violence, because they were seen as an assault on individual freedoms, or even as a political conspiracy (cf. Finkelstein et al., 2020; Shepherd, 2020; Siegler, 2020; Thomson and Ip, 2020). Men were less likely than women to wear masks (Haischer et al., 2020), presumably because they were more likely to view mask as “shameful, not cool, [and] a sign of weakness,” as documented by Capraro and Barcelo (2020), see also Glick (2020). Men's greater reluctance to wear masks was also associated with a lower sense of susceptibility to COVID-19 (Capraro and Barcelo, 2020). This latter finding was ironic in light of men's greater vulnerability to severe consequences from the disease (e.g., Bwire, 2020; Mallapaty, 2020).

### U.S. Government's Communication on Masks

During much of the COVID-19 pandemic in 2020, the United States government lacked a clear direction. Whereas the CDC declared a public health emergency and eventually urged Americans to wear masks, communications from the White House varied, and even contradictory at times. President Trump asserted variously that the virus would go away or that the situation was fully under control [e.g., Gabbatt and Evelyn, 2020; Mangan, 2020; for a summary and timeline of Trump's claims, see Blake (2020) and Wolfe and Dale (2020)]. This occurred as the number of new infections and deaths was multiplying. In early April 2020, President Trump did recommend the voluntary use of masks to stem the infection (Wu and Jackson, 2020); however, he downplayed their urgency and said, “I don't think I'm going to be doing it.” Though public health officials around the country urged citizens to don masks, Trump seemed to resist doing so. When asked why he was not wearing a mask in public, on May 21, he said that he “didn't want to give the press the pleasure of seeing it” (Carlisle, 2020). Many public observers noted a somewhat lax attitude toward masks at the White House. When, after a break of several months, President Trump resumed holding large rallies, many members of his audience did not wear masks (Egan, 2020b). Trump himself first appeared wearing a mask in public on July 11, at least four months into the pandemic (McFall, 2020).

Beyond the reluctance to embrace masks as a cheap, widely available, and effective tool against infection, President Trump himself accused Democrats of using COVID-19 merely as an opportunity to attack and criticize him, emphasizing that he was doing all that was necessary. The President, members of his family, and political pundits repeatedly characterized COVID-19 as a “hoax” which Democrats used during an election year to undermine the U.S. economy and distract from Trump's accomplishments (Egan, 2020a). On May 17, 2020, Eric Trump, the President's son, said: “You watch — they'll milk it every single day between now and November 3. And guess what? After November 3, coronavirus will magically all of a sudden go away and disappear and everybody will be able to reopen. They're trying to deprive him of his greatest asset” (Rupar, 2020). With 2020 being an election year, statements like this quickly led to the politicization of common-sense public health measures, including wearing masks. Conservative supporters of Donald Trump considered many public health measures that limited personal movement and economic activity to be an overreaction at best, but at their worst a deliberate attempt to undermine a sitting president's chances at re-election (e.g., Ingraham, 2020; O'Connell, 2020). Liberal opponents of Donald Trump accused him of persistent mismanagement, an insufficient reliance on facts and science, and simply not taking the pandemic seriously enough—at the expense of Americans' well-being (e.g., Acosta and Vazquez, 2020; Karanth, 2020).

### Mask Wearing as Cultural Behavior

Whereas wearing a mask in public was a novel behavior for most Americans, we argue that masks and the requirement to wear them must be interpreted through existing cultural frameworks [see also Timpka and Nyce (2021)]. With most Americans being unfamiliar with pandemics, we argue that people relied on existing conceptual frames and ideas to arrive at an understanding of masks. Rather than merely focusing on the immediate purpose, we argue that as a public, and publicly argued over behavior, masks took on a meaning that went beyond its immediate purpose of limiting infection. Cloth and procedure masks are primarily useful in limiting the spread of infection by the person wearing the mask (assuming the person carries the virus; Howard et al., 2021). With many infections remaining asymptomatic, and persons being unaware of their infection status, wearing a mask can be viewed as a prosocial act to cooperate in the protection of their loved ones or the community as a whole, even when it implies some personal discomfort (e.g., Cheng et al., 2020; Pfattheicher et al., 2020; Campos-Mercade et al., 2021). For others, wearing a mask is a symbol of government overreach, and the requirement to wear masks is an infringement on personal freedom (e.g., Vuolo et al., 2020). For a third group, the need to wear masks may reflect a concession of weakness (Capraro and Barcelo, 2020). It highlights that the wearer might be a source of infection, and that society as a whole is currently unable to deal with the disease through more advanced means (Goldberg, 2020). Yet, for others wearing a mask is an expression of individuals accepting personal responsibility for doing their part in an otherwise overwhelming crisis (Liu, 2021; Timpka and Nyce, 2021). Acknowledging that masks may be interpreted in a number of divergent ways, we applied three established theoretical frameworks of cultural psychology to the problem. Specifically, we examined masks and mask wearing from the perspective of individualism-collectivism, the tightness-looseness framework, and research on U.S. honor culture.

#### Individualism-Collectivism

Past studies conducted under the broad umbrella of this research distinguish divergent motivational orientation and social ways, in which individuals position themselves vis-à-vis others. Individualistic societies generally champion individual autonomy and uniqueness, where people often assume that individuals are inclined to pursue their self-interest. Conversely, in collectivistic societies, individuals tend to view themselves as part of a collective, with individuals often willing to forgo their self-interest for the benefit of their group (e.g., Triandis, 1995; Oyserman et al., 2002). Whereas these characteristics refer largely to societal distinctions, theorists have long pointed out that different types of societies tend to encourage different cultural beliefs and views of the self. Markus and Kitayama (1991, 2010) proposed a distinction between independent and interdependent self-construal, which individuals from different cultural contexts might embrace to different degrees. Independence refers to viewing the self as an autonomous agent, disconnected from others, but who nevertheless might agree to cooperate with others. Independent individuals tend to be invested in self-expression and their personal choices (e.g., Kim and Markus, 1999; Kim and Sherman, 2007). For independent individuals, considerations of their self-interest often loom large, with individuals often considering the cost and benefits associated with personal choices (e.g., Markus and Kitayama, 1991; cf. Miller, 1999; Oyserman et al., 2002; Utz, 2004). Yet, they might also entail ethical beliefs that highlight individual responsibility (e.g., Waterman, 1981, 1984; cf. Kemmelmeier et al., 2006). Interdependent individuals are more likely to define themselves as a member of a group (Markus and Kitayama, 1991). They are invested in supporting the group and abiding by its norms and requirements, even if this implies subordinating one's personal preferences to that of the group (Triandis, 1995). This implies that interdependent self-construal often entails that the collective interest or the interest of others takes priority over one's immediate self-interest (e.g., LeBoeuf et al., 2010; Savani et al., 2011). At the same time, interdependent individuals may not be strangers to considerations of self-interest. Fjneman et al. (1996) argued that, to the extent that they contribute to a shared collective effort, they also expect to be supported by members of the very same group; in other words, they do expect a return on their investment in the collective.

In the present research, we examined the implications of individualism-collectivism/independence-interdependence both at the societal level and the individual level. In cultural research, it has long been demonstrated that individual-level beliefs and societal-level characteristics do not have to correspond. Characteristics observed for a society as a whole cannot necessarily be reduced to individual characteristics (Na et al., 2010). Moreover, every culture tends to harbor considerable heterogeneity in that individuals may or may not embrace mainstream values, or their specific cultural experiences may be shaped by proximal forces that are different than for many other members of the same society [based on religion, social class, ethnicity, etc.; see, e.g., Coon and Kemmelmeier (2001), Oyserman et al. (2002), Cohen (2009), and Stephens et al. (2014)]. Still, based on the literature we fundamentally expected parallel outcomes regardless of whether we assessed individualism-collectivism (state-level differences) or independence-interdependence (individual differences).

We anticipated that Americans from more collectivistic U.S. states as well as Americans who viewed themselves as more interdependent would have more favorable evaluations of masks and mask wearing. Americans from these types of states should view mask-wearing as normative as they would generally expect members of the community to do what is in the best interest of the community. Likely, the threat of the pandemic would activate a genuine concern among those high in interdependence for the well-being of others, and the well-being of their community. In the language for Janoff-Bulman and Leggatt (2002), for those high in interdependence meeting social expectations (“shoulds”) become a personal desire (“wants”). As a consequence, those high in interdependence might develop more favorable evaluations of mask wearing and its usefulness, the officials who imposed the policy, and potential experience wearing masks as rewarding to the extent that it highlights their commitment to their relationships and community.

Concerning Americans from more individualistic states and those who see themselves as more independent, we did not necessarily expect that they are insensitive to the demands of the historical moment, and insist on not wearing a mask. Rather, we argue that independent individuals are either focused on mask wearing being in their self-interest or a sense of personal responsibility which should also orient them toward prosocial behavior (e.g., Kemmelmeier et al., 2006). However, other than individuals high in interdependence, those high in independence should experience their cooperation as being induced by the requirements of the situation, and not a personal desire. That is, whereas those high in interdependence embrace the goals of the community as their own, those high in independence might be clear that their cooperation does not reflect their personal desire but rather a sense of responsibility, even at a personal cost to them.

Note that the literature generally treats individualism-collectivism as the opposite ends of the same underlying theoretical dimensions (e.g., Vandello and Cohen, 1999; Hofstede, 2001). However, when assessed at the individual level, independence and interdependence tend to constitute orthogonal dimensions (e.g., Singelis, 1994; Taras et al., 2014). This is in part because in the lives of every individual, independent and interdependent aspects of the self may be salient at different moments, with different self-construals being relevant in different situational contexts.

#### Tightness-Looseness

The concept of tightness-looseness describes the overall strength and consensus of social norms and the tolerance of deviance in a given society (Gelfand et al., 2011; Harrington and Gelfand, 2014; Uz, 2015). Tight cultures tend to have stronger norms and are less tolerant of deviance, whereas loose societies have weaker social norms and are tolerant of individuals engaging in unusual and non-normative behaviors. People in tighter cultures are more likely to self-monitor to ensure that they behave in line with accepted norms and standards, and they are more accepting of government action that prevents access to materials and restricts behaviors that are considered untoward.

Recent research on U.S. states has demonstrated the implications of tightness-looseness for infectious disease. Harrington and Gelfand (2014) reported that tighter U.S. states exhibited higher rates of influenza, pneumonia, and various sexually transmitted diseases, which the authors interpreted as support for the notion that external threats, including pathogens, foster tighter societal norms [see Jackson et al. (2020)]. More recent work by Gelfand et al. (2021), however, has argued that tighter societies are more successful at fighting off pandemics, which are much more punctuated events requiring a societal response. Because mitigating COVID-19 requires a great deal of coordination, societies with strong behavioral norms are more likely to succeed at implementing effective non-pharmaceutical interventions, such as wearing face masks and social distancing [see Perra (2021)]. In keeping with this hypothesis, Gelfand et al. (2021) demonstrated lower levels of infection and death in tighter compared to looser societies. Whereas these authors assumed that greater compliance with non-pharmaceutical interventions was the critical causal mechanism, the authors' data were unable to address this question empirically. The present investigation does examine this question directly by examining the correlation between tightness-looseness and mask wearing, both in terms of behavior and attendant attitudes.

Predictions for mask-wearing behavior and perceptions are straightforward. Individuals from tighter cultures should be more willing to comply with official requests to wear masks. Because wearing a mask ultimately reflects them complying with a social norm, they should not find wearing masks personally aversive and approve of government officials who are establishing this norm, and trust their judgment.

#### Honor Culture

Recent research on honor culture has demonstrated that, in part because of their differential immigration history, there are marked differences between U.S. states (e.g., Nisbett and Cohen, 1996; Cohen, 1998). Starting in the American South, and subsequently spreading to other areas of the country, honor cultures emphasize self-reliance and individuals' ability to defend themselves, if necessary, with physical aggression^1^. Indeed, early research on honor culture focused primarily on the elevated patterns of violence (e.g., Gastil, 1971; Nisbett, 1993). Honor cultures tend to arise in economically challenging environments in which the influence of government and law enforcement is relatively weak, forcing individuals to fend for themselves. Because physical altercations are costly, even for the party that prevails, a code of honor relies on the public display of toughness and personal strength, which serves to deter potential aggression against the self [see also Anderson (1994)]. Much of the available research has traditionally emphasized that this is primarily the case for men in honor cultures (e.g., Üskül et al., 2019). Yet, recent research has documented that in honor cultures, women are also ready to engage in violence (e.g., Berthelot et al., 2008) and women holding honor values are motivated to seek retribution against transgressors (cf. McLean et al., 2018; Crowder et al., Unpublished Data).

Individuals in honor cultures tend to be invested in being seen as a “person to be reckoned with.” Masks are likely to present a challenge to this public image that individuals seek to project. Because masks imply a concession that the person is otherwise defenseless against a virus, members of honor cultures should be particularly likely to view masks as a sign of weakness. This should render members of honor cultures reluctant to wear masks. But to the extent that they do wear masks, they should experience this as a loss of social status (Brown, 2016).

#### Political Orientation

As discussed, in the context of the American political landscape, masks, along with other public health measures, quickly became assimilated to the highly polarized political environment, in which those who embraced masks were under the suspicion of being opponents of President Trump, and vice versa. Arguably, one of the reasons for this division is that political groups can also be conceived of as cultural differences (Malka, 2014). Conservatives and liberals tend to embrace different value priorities (Wetherell et al., 2013; Jones et al., 2018) and often diverge in the moral criteria they apply (Haidt and Graham, 2007; Waytz et al., 2019). Likewise, there is evidence that conservatives and liberals exhibit different thinking styles (Talhelm et al., 2015; Yilmaz and Saribay, 2016, 2017). Others have observed a tendency for cultural characteristics to cluster, such that individuals' ideological identification as liberal or conservative increasingly serves as a proxy for the lifestyles they lead (e.g., DellaPosta et al., 2015). With the self-segregation of liberals and conservatives in terms of geography (Motyl et al., 2014) and in the media sphere (Bakshy et al., 2015), there is increasing reason to treat liberalism and conservatism as subcultures within the broader context of American culture (cf. Cohen and Varnum, 2016)^2^.

In the context of the pandemic, liberals tended to emphasize the fact that COVID-19 represented an imminent public health threat, requiring an immediate response by the government and the entire society. Conservatives often considered the pandemic less severe and the response disproportional, with experts being considered less than competent, and public health measures, such as wearing a mask being misguided or ineffective (Calvillo et al., 2020; Conway et al., 2021; Latkin et al., 2021). For many conservatives the fear of an overreaching government response far outweighed their concern with the virus, leaving them to be suspicious of, if not resisting public health directives.

In the present investigation, we expected conservatism to be related to lower levels of mask wearing, and less favorable evaluation of masks and masking in general. In line with previous research (e.g., Rudolph and Evans, 2005), we expected conservatives to have lower trust in government and perceive masks primarily in terms of the limitation that they imposed on individuals.

#### The Present Study

The goal of our study was to explore mask wearing as cultural behavior within the broad cultural frameworks described above. The simultaneous investigation of different cultural predictors allowed us to identify the unique contributions of each; it also enabled us to examine whether different facets of masks and mask wearing would be subject to similar or different cultural forces. Our interest focused on past mask-wearing behavior, future intents, but also the possibility of respondents changing their behavior. We further examined respondents' beliefs about the utility of mask wearing and its effectiveness in reducing infections, how normative and socially expected mask wearing was perceived to be, but also examined how much trust respondents placed in the public officials who issued mask mandates or related recommendations. A critical aspect of our work was an examination of the symbolic meaning of masking. We conducted a survey that included respondents from different U.S. states. Using multilevel modeling, we predicted survey responses based on individuals' cultural beliefs as well as the cultural characteristics of the different states, in which these individuals resided. At the individual level, we investigated the implications of self-reports of independent and interdependent self-construals, as well as conservatism. To examine the effects of cultural contexts, we relied on state-level predictors of individualism-collectivism, tightness-looseness, and honor culture. In all of our analyses, we controlled for whether masks were mandated in respondents' community, gender, education, age, ethnicity at the individual level, and wealth and social inequality at the U.S. state level.

## Methods

### Respondents

Our sample consisted of 633 respondents (40% female; 77% White) from 45 states. Sample sizes per state varied from a minimum of 6 (e.g., Oregon, Rhode Island) to 55 (California; see Supplementary Material S1 for details). On average, the sample included 14.1 respondents per state (*Md* = 9). Respondents had taken an average of 350 seconds to participate (*SD* = 189; *Md* = 310 s; range 121–2,367 s). Our Supplementary Material S1 provides a detailed discussion of power considerations, recruitment, data cleaning, and sample characteristics.

### Measures and Procedure

#### Dependent Variables

Unless stated otherwise, respondents answered all items on a five-point scale ranging from 1 *Strongly disagree* to 5 *Strongly agree*, with the midpoint 3 labeled *Neither agree nor Disagree*. Variables were combined to the extent that they were both theoretically coherent and substantially correlated. We did not combine variables that were not substantially correlated, even when we had generated them in hopes to form a scale. All items are listed as part of our Supplementary Material S2.

##### Mask-Wearing Behavior

One question tapped past behavior, with respondents choosing one of five responses: *Never, Sometimes, About half the time, Most of the time*, and *Always*. Another item referred to future intent.

##### Behavior Change

Two items sought to capture to what extent respondents felt that they would be responsive to the social instigation of others^3^. Because items were highly correlated (*r* = 0.82), they were collapsed into one index.

##### Knowledge

Respondents indicated their knowledge about the virus using one item (“How much do you feel you know about the novel coronavirus?”). Respondents described themselves on a 5-point scale ranging from 1 *Not knowledgeable at all* to 5 *Extremely knowledgeable*.

##### Mask Utility

Seven questions asked whether it was in different parties' interests to wear a mask. Two questions referred to self-interest and another two tapped others' interests. Three questions addressed to which extent respondents believed wearing masks to be effective. Though originally conceived to address different beliefs, the seven items were highly correlated, Cronbach's α = 0.87, and grouped around a single factor, MacDonald's ω = 0.80, and were therefore combined^4^.

##### Feeling of Protection

Because masks may make others feel protected, we included two questions on issue. Because of their substantial correlation, *r* = 0.50, the items were combined.

##### Social Norms

Three questions tapped social expectations concerning mask wearing. Combined into one index, the reliability was α = 0.74.

##### Social Recognition

Two questions inquired whether respondents felt recognized by others. Because they were highly correlated, *r* = 0.71, these two items were combined.

##### Trust in Officials

Two questions tapped the extent that respondents trusted public officials with regards to the necessity of wearing masks. The first item referred to health professionals, whereas the second item referred to elected officials. The latter item was reversed such that for both items higher values indicated higher levels of trust. Because these items were only moderately related, *r* = 0.30, they were analyzed separately.

##### Negative Evaluation

Two items addressed how respondents felt about wearing masks and their necessity. Because of their substantial correlation (*r* = 0.49), these items were collapsed into one index.

##### Social Image

Two questions addressed the extent to which respondents experienced mask wearing as undermining a favorable appearance. The first item was related to strength, and the second item implied that masks undermined the mask wearer's social standing in the eyes of non-benevolent others. Although these items were substantially correlated, they were analyzed separately to allow comparison with other research that had specifically focused on perceptions of weakness, and to retain the specific content that had sparked this item^5^.

##### Low Well-Being

A total of four items inquired to what extent wearing a mask induced negative feelings. These four items were highly correlated, Cronbach's α = 0.88 and MacDonald's ω = 0.84, and were subsequently combined.

##### Freedom vs. Civic Duty

One item captured to what extent respondents viewed wearing a mask as a limitation on their freedom, and another one asked whether it represented a civic duty. A third item tapping principled opposition to mask wearing turned out to be closely aligned with the first item, and was therefore included in this three-item index, α = 0.74. Higher values indicated that wearing a mask represented an infringement.

##### Voluntariness

One question sought to capture whether mask wearing is experienced as a mandate or a voluntary and presumably prosocial act.

#### Cultural Differences Between Individuals

##### Self-Construals

We used a set of 15 items to assess independent, group-interdependent, and relational-interdependent self-construals using five items each. Merhi (2021) selected these items from a set of 73 items based on five previously published scales. Specifically, Merhi (2021) employed the Delphi method (e.g., Dalkey and Helmer, 1963) to distill a subset of items in an iterative process involving an international group of experts in cultural psychology. All three self-construal scales were reliable: independence α = 0.74, collective interdependence α = 0.82, and relational interdependence α = 0.80. See Supplementary Material S2 for sample items; for complete scales see Merhi (2021).

##### Political Orientation

Respondents were asked to locate their political views on a five-point left-to-right scale, and also describe them on a five-point liberal-to-conservative scale. These two items were highly correlated, *r* = 0.82, and combined into one index with higher values indicating higher levels of conservatism.

#### Individual-Level Control Variables

##### Gender

Respondents described themselves as either male, female or other (with an opportunity for them to elaborate).

##### Age Group

Respondents were asked to classify their age as being between 18 and 24, 25 and 34, 35 and 44, 45 and 54, 55 and 64, 65 and 74, or as 75 and older.

##### Ethnicity

Respondents were asked to describe themselves as either White or European American, Asian/Native Hawaiian or Pacific Islander, Black or African American, Hispanic or Latino, or other, with an opportunity to provide a more detailed description.

##### Education

Respondents recorded if they had not completed high school, if they had completed high school/GED, whether they had some college, a bachelor's degree or if they had completed advanced graduate work.

#### Local Government Policy Concerning Masks

We asked respondents as to whether or not in their jurisdiction there was an order in place requiring the wearing of a mask in public places. Though this variable was technically assessed at the individual level, it does pertain to respondents' community.

#### Cultural Differences Between U.S. States

##### Collectivism

To characterize a state's culture in terms of individualism and collectivism, we relied on the index proposed by Vandello and Cohen (1999), which was based on eight variables tapping social and residential structures (e.g., living arrangements, divorces). Scores ranged from 31 (*least collectivist/ most individualist*) to 72 (*most collectivist/least individualist*). The authors reported a standardized Cronbach's α of 0.71 for different between-state variables. See Supplementary Material S3 for additional details.

##### Tightness-Looseness

We used the tightness-looseness score proposed by Harrington and Gelfand (2014). Following the method originated by Vandello and Cohen (1999), the authors generated and validated an index based on nine variables characterizing differences between states (α = 0.84). See Supplementary Material S3 for additional details.

##### Honor Culture

Based on the analysis of Cohen (1998), each state was coded as to whether it represented an honor culture or not based on the state's history and economics, and also considered migration patterns from the U.S. South to other states [0 = No, 1 = Yes; see also Nisbett and Cohen (1996)].

#### State-Level Control Variables

##### State Wealth

We accounted for state differences in general state product per capita (GSP) which were obtained from the Bureau of Economic Analyses (2020).

##### State Inequality

We used the 2021 Gini index for each state, calculated based on data from the American Community Survey (2021; see also https://worldpopulationreview.com/state-rankings/income-inequality-by-state). For an alternative set of analyses, we obtained state poverty levels for 2019 from the U.S. Census (https://www.census.gov/quickfacts/US); because state poverty levels did not qualify any of our findings, our main analyses included the Gini index as a measure of social inequality.

##### Additional State-Level Covariates

Various analyses also controlled for the median age of each state because age represents a critical risk factor for severe consequences of COVID-19, we also included the median age of each state as a predictor. Likewise, we examined latent political differences between states by controlling for the share of state legislators who were members of the Democratic Party. Lastly, to gauge the level of threat in a state at the time of our survey, we controlled for the total number of COVID-19 cases in a state, as well the share of the population affected by the disease. Because none of these additional predictors qualified our results, they are not reported further in the main text (see variable details in Supplementary Material S4).

## Results

### Descriptive Analyses

#### Individual-Level Dependent Variables

Table 1 displays the mean, standard deviations and zero-order correlations of all dependent variables assessed in the present study. Overall, in the present sample respondents indicated a fairly high level of mask-wearing behavior, with most respondents saying that they wore masks most of the time (*M* = 4.24; *Md* = 5), a finding broadly consistent with other data showing high levels of mask wearing in the U.S. in summer 2020 [but see Blakemore (2020), e.g., Hutchins et al. (2020)]^6^. Expressed intent to wear a mask was equally high, with the mean falling squarely in between the *Agree* and the *Strongly agree* response (*M* = 4.41; *Md* = 5). Both past behavior and future intent were correlated with the perception that the utility of wearing masks is high, and that they would make others feel protected. Past behavior and future intent were also linked to respondents viewing it to be a civic duty rather than an infringement on their freedom, and the perception that mask wearing was normative. Unsurprisingly, people who wore masks more frequently in the recent past or who intended to wear masks in the future attributed fewer negative consequences to masks than people who did not wear masks previously and had no intention of doing so in the future.

Table 1Correlation matrix and descriptive statistics for individual-level variables (dependent variables).

**Table:** 

**Scale**	**(2)**	**(3)**	**(4)**	**(5)**	**(6)**	**(7)**	**(8)**	**(9)**	**(10)**	**(11)**	**(12)**	**(13)**	**(14)**	**(15)**	**(16)**
(1) Mask-wearing behavior	0.64	−0.09	0.05	0.56	0.45	0.36	0.25	0.17	0.29	−0.34	−0.26	−0.19	−0.27	−0.38	−0.05
(2) Mask Wearing intent		−0.08	0.02	0.63	0.50	0.41	0.23	0.24	0.28	−0.36	−0.35	−0.27	−0.34	−0.44	−0.06
(3) Behavior change			0.26	−0.02	0.04	0.07	0.35	−0.20	0.19	0.39	0.46	0.53	0.52	0.44	0.39
(4) Coronavirus Knowledge				0.06	0.13	0.13	0.33	−0.16	0.18	0.20	−0.40	−0.28	−0.35	−0.61	0.02
(5) Mask Utility					0.68	0.46	0.38	0.30	0.48	−0.47	−0.40	−0.28	−0.35	−0.61	0.02
(6) Mask Protects others						0.50	0.45	0.20	0.50	−0.32	−0.22	−0.13	−0.22	−0.44	0.07
(7) Social Norms							0.30	0.18	0.38	−0.11	−0.12	−0.03	−0.09	−0.23	−0.24
(8) Social Recognition								−0.05	0.41	−0.01	0.23	0.33	0.24	0.05	0.29
(9) Trust Government Officials									0.30	−0.46	−0.54	−0.46	−0.49	−0.52	−0.32
(10) Trust Public Health										−0.18	−0.03	−0.10	−0.28	0.10	−0.03
(11) Negative Evaluation											0.69	0.57	0.71	0.73	0.24
(12) Sign of weakness												0.76	0.77	0.78	0.42
(13) Not wanting to be seen with a mask													0.76	0.72	0.42
(14) Low Well-being														0.75	0.42
(15) Freedom vs. Civic Duty															0.34
(16) Voluntariness

**Table:** 

	**(1)**	**(2)**	**(3)**	**(4)**	**(5)**	**(6)**	**(7)**	**(8)**	**(9)**	**(10)**	**(11)**	**(12)**	**(13)**	**(14)**	**(15)**	**(16)**
Mean	4.24	4.41	3.12	3.71	4.27	4.12	4.08	3.35	2.84	3.88	2.80	2.29	2.45	2.72	2.32	3.20
*SD*	0.99	0.92	1.34	0.85	0.77	0.79	0.76	1.13	1.40	1.10	1.27	1.52	1.51	1.26	1.16	1.27

*Pearson correlations r > 0.09 are significant at p < 0.05*.

Whereas there are many other correlations worth commenting on, we limit ourselves to a few notable observations. The view that mask wearing was not necessary was correlated much more strongly with their perceived symbolic meaning than with utilitarian purposes (e.g., if mask wearing protects community). Specifically, the view that masks were unnecessary and aversive was tightly linked to the assessment that mask wearing was an infringement on one's freedom (*r* = 0.73) and that it was a sign of weakness (*r* = 0.69). Similarly, to the extent that masks represented a sign of weakness, respondents agreed with the item that they did not want to give others the satisfaction of seeing them with a mask, they attributed low well-being consequences to masks (both *r* = 0.77), and considered wearing masks an infringement rather than a civic duty (*r* = 0.78). This pattern corroborates the notion that controversies in the U.S. over the necessity of masks, as they took place during much of 2020, were fought to a large extent because masks symbolized very different things to Americans based on different cultural frameworks.

Similarly, the view that masks represented normative behavior was correlated with respondents' own past behavior (*r* = 0.36), and their higher utilitarian value (*r* = 0.46). Whether masks were perceived to constitute normative behavior was only weakly correlated with one's own negative personal evaluations (negative evaluations; *r* = −0.11), and weakly linked to negative consequences on one's well-being (*r* = −0.09). Rather, individuals who viewed mask wearing as normative seemed to derive social recognition from wearing masks themselves (*r* = 0.30).

Lastly, it was striking how clearly respondents distinguished between government officials and public health officials. Table 1 reveals that there were more substantial correlations between mask-wearing behavior and the utility of masks, on the one hand, and trust in public health officials, on the other hand (*r* = 0.29 and *r* = 0.48, respectively), than there were correlations between these two variables and trust in government officials (*r* = 0.17 and *r* = 0.30, respectively). Most tellingly, perceptions of masks as aversive, and unnecessary, symbols of weakness and a threat to one's public image were strongly linked to distrust in government (*r* = −0.46, −0.54, and −0.46, respectively), whereas being only weakly related to higher trust in public health officials (*r* = −0.18, −0.03, and −0.10, respectively).

#### Individual-Level Predictors

Means, standard deviations, and intercorrelations are summarized in Table 2A. Confirming expectations, the two interdependence dimensions were strongly correlated, though not redundant with each other, *r* = 0.60. As observed in previous work, there was only a weak or no correlation between independent and interdependent self-construals (e.g., Taras et al., 2014). The present research also confirmed past reports by Kemmelmeier et al. (2003) that, in the U.S., conservatism was unrelated to independence, but positively related (*albeit* weakly) to interdependence. Associations with ethnicity were generally not significant, with few exceptions (see Supplementary Material S5).

**Table 2A:** Correlation matrix and descriptive statistics for individual-level variables.

**Scale**	***M***	**(*SD*)**	**(2)**	**(3)**	**(4)**	**(5)**	**(6)**	**(7)**	**(8)**	**(9)**	**(10)**
(1) Independence	4.19	(0.49)	0.09	0.20	−0.05	0.07	−0.02	0.07	−0.06	−0.04	−0.10
(2) Coll. Interdepend.	3.82	(0.71)		0.60	0.17	0.02	−0.03	0.14	0.01	0.07	0.16
(3) Rel. Interdepend.	3.92	(0.73)			0.14	0.02	−0.10	0.08	−0.02	0.08	0.10
(4) Conservatism	3.10	(1.33)				−0.05	−0.14	−0.01	−0.03	−0.04	0.18
(5) Gender	0.42						0.00	−0.08	−0.05	0.08	0.03
(6) Asian (vs. white)	0.05							–	–	–	−0.02
(7) Black (vs. white)	0.12								–	–	0.03
(8) Hispanic (vs. white)	0.05									–	0.01
(9) Other (vs. white)	0.01										−0.08
(10) College or higher	0.74										

*N = 633 (lower in case of missing data and for ethnic variables)*.

*Pearson correlations >0.09 are significant at p < 0.05. Variables (6) through (9) refer to comparisons between whites and one other ethnic group only*.

#### State-Level Variables

Table 2B displays the means, standard deviation of our state-level predictors. The first five variables reflect genuine state-level predictors (1–5), whereas the latter four variables represent the state-level averages of our individual-difference predictors (6–9). Across the 45 U.S. states included in the present study, we observed a moderate-sized correlation between honor culture and tightness-looseness, consistent with Harrington and Gelfand (2014). This was also the case for GSP, which correlated strongly with both honor culture and tightness-looseness at the state level, though inequality (as captured by the Gini index) was modestly related to tightness and collectivism, but also to GSP. Notably, state-level collectivism was weakly linked to all other state-level variables, though most strongly associated with tightness [see also Harrington and Gelfand (2014)]. Yet, there was a convergence of Vandello and Cohen's (1999) collectivism index and the state-level averages of collective and relational interdependence. The fact that the size of the correlations still amounted to <25% of the variance in both variables corroborates that cultural analysts must distinguish between predictors at the individual level and the societal level (Na et al., 2010). Remarkably, state averages in conservatism-liberalism were not only correlated with all three state-level culture variables (honor, tightness, and collectivism), but also with state averages in collective interdependence [see Kemmelmeier et al. (2003)]^7^.

**Table 2B:** Correlation matrix and descriptive statistics for state-level variables.

**Scale**	***M***	**(*SD*)**	**(2)**	**(3)**	**(4)**	**(5)**	**(6)**	**(7)**	**(8)**	**(9)**
(1) Honor	0.58	(0.50)	0.32	0.13	−0.40	−0.15	0.25	−0.01	0.08	0.36
(2) Tightness	50.61	(13.02)		0.14	−0.59	0.18	0.14	0.11	0.20	0.39
(3) Collectivism	50.73	(11.51)			−0.03	0.28	−0.14	0.38	0.29	0.27
(4) GSP	61031	(11556.66)				0.14	−0.31	0.01	−0.17	−0.25
(5) Gini	47.18	(1.94)					−0.05	−0.24	−0.23	−0.34
(6) Independence	4.20	(0.17)						−0.09	0.01	0.07
(7) Collective Interdependence	3.79	(0.24)							0.45	0.48
(8) Relational Interdependence	3.89	(0.24)								0.25
(9) Conservatism	3.10	(0.49)								

*K = 45. Pearson correlations >0.29 are significant at p < 0.05. GSP stands for General State Product per capita*.

### Multilevel Regression Analyses

#### Analytical Approach

We submitted all dependent variables to a two-level mixed-effects (multilevel) regression model in which respondents were treated as nested within U.S. states. At the individual level (level 1) we entered demographic information as predictors as well as individual differences in culture orientation (self-construals and conservatism). These variables also included respondents' reports as to whether mask wearing was mandatory in their jurisdiction. At the state level (level 2), we initially added the critical predictors: honor culture, collectivism, and tightness-looseness, as well as GSP and the Gini coefficient as covariates. All predictors were modeled as fixed effects, with all continuous predictors being grand-mean centered (see Supplementary Material S6 for the regression equation). All multilevel analyses were conducted in R using the *lmer* function of the *lme4* package (Bates et al., 2015).

All results are summarized Table 3 through **Table 6**. In the bottom section, we report indicators of model fit as well as the intra-class correlation (ICC) of the null model to convey what share of variance occurred between states rather than between individuals. Note that the ICCs of our dependent variables were small, ranging from 0 to 0.054. Hence, the *a priori* likelihood of detecting any between-states differences was low, simply because Mturk respondents from different states did not seem to differ very much from each other.

**Table 3:** Result of multilevel analyses: mask-wearing behaviors, behavior change, and knowledge.

	**Mask wearing behavior**		**Mask wearing intent**		**Behavior change**		**Coronavirus knowledge**	
	***b***	**(*se*)**	***b***	**(*se*)**	***b***	**(*se*)**	***b***	**(*se*)**
Intercept	3.84^***^	(*0.23*)	4.30^***^	(*0.20*)	2.77^***^	(*0.32*)	3.27^***^	(*0.19*)
**Individual-level**								
Female (Male = 0)	0.14^+^	(*0.08*)	−0.03	(*0.07*)	−0.21^*^	(*0.09*)	−0.05	(*0.07*)
Education (High School = 0)								
Some college	0.21	(*0.17*)	0.06	(*0.15*)	0.30	(*0.19*)	0.10	(*0.14*)
College	0.15	(*0.16*)	< -0.01	(*0.14*)	0.74^***^	(*0.18*)	0.40^**^	(*0.13*)
Advanced Deg.	0.30^+^	(*0.18*)	0.01	(*0.16*)	0.71^***^	(*0.20*)	0.48^***^	(*0.14*)
Age (18–24 years = 0)								
25–34	0.09	(*0.17*)	−0.03	(*0.15*)	−0.11	(*0.19*)	−0.06	(*0.14*)
35–44	−0.07	(*0.18*)	−0.11	(*0.16*)	−0.14	(*0.20*)	−0.11	(*0.14*)
45–54	−0.06	(*0.19*)	−0.03	(*0.17*)	−0.10	(*0.22*)	−0.16	(*0.16*)
55–64	−0.01	(*0.22*)	−0.29	(*0.20*)	−0.11	(*0.26*)	−0.01	(*0.18*)
65–74	0.30	(*0.34*)	−0.03	(*0.30*)	−0.25	(*0.38*)	−0.37	(*0.28*)
Race/Ethnicity (White = 0)								
Asian	0.23	(*0.18*)	0.24	(*0.17*)	< -0.01	(*0.21*)	−0.07	(*0.15*)
Black	0.21	(*0.13*)	0.14	(*0.12*)	−0.13	(*0.15*)	−0.04	(*0.11*)
Latinx	0.08	(*0.19*)	−0.04	(*0.17*)	−0.10	(*0.22*)	0.08	(*0.16*)
Other	0.39	(*0.44*)	0.41	(*0.40*)	−0.36	(*0.50*)	0.02	(*0.36*)
Independence	0.13	(*0.07*)	0.24^***^	**(*****0.07*****)**	−0.18^*^	**(*****0.08*****)**	0.03	(*0.06*)
Collective interdep.	0.13	(*0.07*)	0.20^**^	**(*****0.06*****)**	0.54^***^	**(*****0.08*****)**	0.09	(*0.06*)
Relational interdep.	0.11	(*0.07*)	0.16^*^	**(*****0.06*****)**	–0.07	(*0.08*)	0.16^**^	**(*****0.06*****)**
Conservatism	−0.14^***^	**(*****0.03*****)**	−0.16^***^	**(*****0.03*****)**	0.35^***^	**(*****0.04***)	–0.06^*^	**(*****0.03*****)**
Mask Mandatory	0.13	(*0.11*)	0.19^*^	**(*****0.09*****)**	0.29^*^	**(*****0.12*****)**	0.25^**^	**(*****0.09*****)**
Mask-wearing behavior					–0.08	(*0.05*)		
**State-level**								
Honor State (No = 0)	−0.022	(*0.099*)	−0.036	(*0.086*)	0.151	(*0.118*)	0.057	(*0.075*)
Tightness	0.005	(*0.005*)	0.007	(*0.004*)	−0.010^+^	(*0.006*)	0.002	(*0.003*)
Collectivism	0.011^*^	**(*****0.005*****)**	0.005	(*0.004*)	< -0.001	(*0.006*)	−0.001	(*0.004*)
GSP	−0.002	(*0.005*)	−0.002	(*0.005*)	0.002	(*0.006*)	0.004	(*0.004*)
Gini	0.015	(*0.027*)	−0.003	(*0.003*)	−0.005	(*0.003*)	0.001	(*0.021*)
**Variance components**								
State		0.01		0.00		0.02		0.01
Residual		0.92		0.74		1.19		0.62
**Model fit**								
AIC		1778.98		1653.94		1928.07		1553.31
BIC		1893.48		1768.39		2046.84		1668.84
−2 Log Likelihood		1726.98		1601.94		1874.08		1501.31
ICC		0.03		0.02		0.03		0.00
Marginal *R*^2^		0.11		0.16		0.34		0.14
Conditional *R*^2^		0.12		0.16		0.36		0.14
*N*		604		603		601		605

+ *p < 0.10*,

* *p < 0.05;*

** *p < 0.01;*

*** *p < 0.001*.

*For our main predictor variables, coefficients and their standard errors are bolded, if they are significant at a minimum of p < 0.05*.

The marginal *R*^2^ refers to the proportion of variance explained by the fixed effects in the model, and the conditional *R*^2^ reflects the proportion of variance explained by both fixed and random effects (Nakagawa and Schielzeth, 2013). Across all models, both parameters varied considerably, ranging from 0.11 to 0.41.

#### Mask-Wearing Behavior

##### Past Behavior

Table 3 summarizes the multilevel regression in which we predicted respondents' mask-wearing behavior (see column 1). Remarkably, whether respondents wore masks in the recent past or not was unrelated to almost all predictors—except for two. Consistent with the politicization of masks, conservatives reported having worn a mask less often than liberals. At the same time, respondents from states scoring higher on collectivism reported more frequent mask wearing, regardless of respondents' personal beliefs concerning independence and interdependence. Still, past mask wearing was unrelated to the variables identified in established cultural frameworks, and it was also unrelated to whether masks were mandatory in respondents' community or not.

##### Intent

However, future intent was more closely tied to cultural frameworks (see Table 3, column 2). Those high in collective interdependence and those high in relational independence were more likely to report that they intended to wear a mask in the future. Higher independent self-construal was also positively related to the intent of mask wearing. Conservatism was negatively linked to the intent of wearing a mask, consistent with their past behavior. Whether mask wearing was mandatory in respondents' community did predict intent, implying that, irrespective of any previous behavior, many respondents intended to wear a mask in the future. Interestingly, none of the state-level predictors was statistically reliable.

##### Behavior Change

The analysis of this dependent variable included one additional predictor, namely self-reported past behavior, thus holding this variable constant (see Table 3, column 3). Those with high levels of independence did reject the idea that they would change their behavior in light of others' expectations. Though people with independent self-construals were not any more or less likely to wear masks, they were intent on doing so in the future; hence, their refusal to respond to social pressures might reflect their dedication to their personal decisions.

In keeping with collective interdependence indicating a motivation to fit in with one's group, respondents scoring high on this dimension expressed a willingness to change their mask-wearing behavior if relevant others wanted them to do (see Table 3, column 3). Notably, this was also the case for conservatism—a finding that must be understood in the context of conservatives being a group to report that they do *not* wear masks and that they have no intentions to do so in the future. Hence, it may appear that conservatives are open to changing their behavior, if those immediately around them request them to do so.

To examine this possibility, in a set of follow-up analyses we only selected those 303 respondents who had said that they wore masks every time they left their house (past behavior score of 5). This analysis equated conservatives and liberals based on past behavior and ensured that any behavioral change would imply a reduction in the behavior. Applying our multilevel model to this subsample, identical effects emerged for collective interdependence, *b(se)* = 0.47(0.12), *p* < 0.001, and conservatism, *b(se)* = 0.44(0.05), *p* < 0.001). Because current mask-wearing behavior was “all the time,” any willingness to change one's behavior can only indicate a lowering in the frequency of mask wearing. Thus, both conservatives and those high in interdependence were responsive to their community and close others, but they were also willing to deviate from what most public health officials considered an urgent need at the time. In an additional step, we only selected those 96 respondents who said that they wore a mask *Never* to *About half the time* (past behavior scores of 1, 2, or 3). In this subsample, conservatism was no longer a significant predictor of willingness to change one's mask-wearing behavior, *b(se)* = 0.01(0.11), *p* = 0.92, whereas collective interdependence remained reliable, *b(se)* = 0.82(0.18), *p* < 0.001. This implies that conservatives only expressed a greater willingness to *reduce* mask wearing compared to liberals, but not to increase it.

#### Knowledge

Though comparatively weak, conservatives were more likely to say that they possessed little knowledge about the novel coronavirus—a surprising observation in light of their apparent resistance to wearing masks (see Table 3, column 4).

As could be expected, if mask wearing was mandatory in their community, respondents were better informed about the coronavirus, presumably because the pandemic was a more pressing issue in their community. Also, those high in relational interdependence reported a higher level of coronavirus knowledge, potentially reflecting their desire to protect people close to themselves.

#### Perceived Utility

In terms of perceived utility, our model demonstrated that collective interdependence predicted that respondents perceived it to be in everyone's interest to wear masks, with also independence being related to a higher perceived utilitarian value (see Table 4, column 1). At the same time, conservatism was related to lower levels of perceived utility. Surprisingly, relational interdependence, which we expected to have a greater sense of caring for close others, was unrelated to this variable.

**Table 4:** Result of multilevel analyses: perceived utility, effects on others, social norms, and recognition.

	**Perceived utility**		**Makes others feel protected**		**Social norms**		**Social recognition**	
	***b***	**(*se*)**	***b***	**(*se*)**	***b***	**(*se*)**	***b***	**(*se*)**
Intercept	4.38^***^	(*0.15*)	4.14^***^	(*0.16*)	3.91^***^	(*0.16*)	2.53^***^	(*0.22*)
**Individual-level**								
Female (Male = 0)	−0.10^+^	(*0.06*)	−0.05	(*0.06*)	0.02	(*0.06*)	−0.12	(*0.08*)
Education (High School = 0)								
Some college	−0.02	(*0.11*)	−0.03	(*0.12*)	−0.13	(*0.12*)	0.35^*^	(*0.16*)
College	−0.03	(*0.11*)	−0.07	(*0.11*)	−0.14	(*0.11*)	0.56^***^	(*0.15*)
Advanced Deg.	−0.03	(*0.12*)	−0.14	(*0.12*)	−0.21^+^	(*0.12*)	0.64^***^	(*0.17*)
Age (18–24 years = 0)								
25–34	0.01	(*0.11*)	0.08	(*0.12*)	−0.06	(*0.12*)	0.16	(*0.16*)
35–44	−0.05	(*0.12*)	−0.03	(*0.13*)	−0.03	(*0.12*)	−0.13	(*0.17*)
45–54	−0.08	(*0.13*)	−0.01	(*0.14*)	−0.02	(*0.13*)	−0.11	(*0.19*)
55–64	−0.13	(*0.15*)	−0.05	(*0.16*)	−0.06	(*0.16*)	0.04	(*0.21*)
65–74	−0.21	(*0.23*)	−0.23	(*0.24*)	−0.34	(*0.23*)	−0.21	(*0.32*)
Race/Ethnicity (White = 0)								
Asian	−0.05	(*0.12*)	−0.05	(*0.13*)	0.14	(*0.13*)	−0.20	(*0.18*)
Black	0.13	(*0.09*)	0.26^**^	(*0.09*)	0.05	(*0.09*)	0.38^**^	(*0.12*)
Latinx	0.01	(*0.13*)	−0.05	(*0.14*)	−0.06	(*0.13*)	0.22	(*0.19*)
Other	0.35	(*0.30*)	0.12	(*0.31*)	0.49	(*0.30*)	0.44	(*0.42*)
Independence	0.18^***^	**(*****0.05*****)**	0.17^**^	**(*****0.05*****)**	0.28^***^	**(*****0.05*****)**	−0.09	(*0.07*)
Collective interdep.	0.38^***^	**(*****0.05*****)**	0.32^***^	**(*****0.05*****)**	0.21^***^	**(*****0.05*****)**	0.46^***^	**(*****0.07*****)**
Relational interdep.	0.08^+^	(*0.05*)	0.23^***^	**(*****0.05*****)**	0.13^*^	**(*****0.05*****)**	0.28^***^	**(*****0.07*****)**
Conservatism	−0.22^***^	**(*****0.02*****)**	−0.11^***^	**(*****0.02*****)**	–0.04^*^	**(*****0.02***)	0.05	(*0.03*)
Mask Mandatory	0.03	(*0.07*)	0.08	(*0.07*)	0.46^***^	**(*****0.07*****)**	0.41^***^	**(*****0.10*****)**
**State-level**								
Honor State (No = 0)	−0.099	(*0.067*)	−0.081	(*0.064*)	−0.075	(*0.082*)	−0.019	(*0.097*)
Tightness	0.005	(*0.003*)	0.001	(*0.003*)	0.004	(*0.004*)	−0.007^+^	(*0.004*)
Collectivism	0.007^*^	**(*****0.003*****)**	0.007^*^	**(*****0.003*****)**	0.008^*^	**(*****0.004*****)**	0.011^*^	**(*****0.005*****)**
GSP	−0.002	(*0.004*)	<0.001	(*0.003*)	−0.004	(*0.004*)	−0.002	(*0.005*)
Gini	−0.020	(*0.019*)	0.024	(*0.017*)	−0.045	(*0.023*)	−0.014	(*0.008*)
**Variance components**								
State		0.01		0.00		0.02		0.01
Residual		0.42		0.46		0.43		0.85
**Model fit**								
AIC		1320.34		1367.40		1329.82		1733.72
BIC		1434.83		1481.76		1443.97		1848.21
−2 Log Likelihood		1268.34		1315.40		1277.82		1681.72
ICC		0.03		0.00		0.02		0.05
Marginal *R*^2^		0.31		0.27		0.24		0.34
Conditional *R*^2^		0.31		0.27		0.28		0.35
*N*		604		601		596		602

* *p < 0.05;*

** *p < 0.01;*

*** *p < 0.001*.

*For our main predictor variables, coefficients and their standard errors are bolded, if they are significant at a minimum of p < 0.05*.

Note that our perceived utility variable combined perceived self-interest and other-interest, not only for respondents themselves to wear masks, but also for others to wear masks. Therefore, we followed up with a series of analyses, which treated each of the seven items as dependent variables in separate analyses. The analyses, reported in full as part of our Supplementary Materials, did confirm the pattern of findings as displayed in Table 4, column 1. Three additional results of interest emerged concerning whether it was in the “community's interest” or “others' interest” for the respondent to wear a mask (see Supplementary Tables S7.1, S7.2, Supplementary Material S7). Regardless of their personal beliefs, respondents from honor-culture states were less likely to agree with both of these items, both *b* = −0.17, *p* < 0.05, suggesting that others' concerns were less important to these respondents. Conversely, respondents from tighter states were more likely to agree with these items, *b* < 0.007, *p* < 0.05, and *b* = 0.008, *p* = 0.054, respectively. The very same items also revealed a similar pattern for state-level collectivism, such that respondents from more collectivistic states were somewhat more likely to say that it is in others interest for them to wear masks, *b* = 0.08, *p* = 0.03, and *b* = 0.007, *p* = 0.068, respectively. Apparently, a cultural emphasis on falling in line with social norms as well as a collectivistic emphasis on community rendered others' interests more salient.

#### Feelings of Protection

Table 4, column 2 summarizes the findings for respondents' belief that wearing a mask conveys a feeling of protection to others. Note that this idea is tied to the perceived utility of masks at preventing infection, but not redundant with it, as it highlights that masks may be taken as a signal to others. Consistent with this notion, we observed a difference. As with perceived utility, high levels of independence, and collective interdependence were positively related to masks conveying a sense of protection, arguably reflecting greater concern for the well-being of one's community and close others. This conclusion was also supported by the observation that, regardless of respondents' personal beliefs, those from collectivistic states were more likely to agree that masks signal a sense of protection to others. Whereas conservatives were less likely to agree with this idea, individuals high in relational interdependence also concurred that masks make others feel protected.

#### Mask Wearing as Normative Behavior

As expected, respondents high in collective or relational interdependence viewed mask wearing to be normative (see Table 4, column 3). That is, to the extent that others wear masks and hold these expectations of everybody in their circle, those high in interdependence do regard mask wearing as the social norm. This pattern was complemented by the fact that respondents from more collectivistic states were also more inclined to view wearing masks as normative. However, higher levels of independence were equally linked to the perception that mask wearing represented a normative behavior. Whereas individualists tend to emphasize individuality, we should not forget that in such contexts, group members expect others to fall in line with individualistic values and norms (e.g., Jetten et al., 2006). Thus, individuals high in independence might view mask wearing as the result of people taking personal responsibility; to the extent that taking personal responsibility is seen as an expectation that is applied to all ingroup members, wearing a mask might be seen as normative among those high in independence as it is for those high in interdependence. Yet, conservative respondents rejected that masks represented a normative behavior.

Comfortingly, wearing a mask was seen as a normative behavior by those who live in jurisdictions in which government officials had mandated mask wearing. This suggests that respondents thought that following the rules set by a government official meant to comply with widely held social expectations.

#### Social Recognition

Respondents high in interdependence indicated that they received recognition from other people when wearing a mask—consistent with the notion that behaving in socially cooperative ways is inherently socially rewarding to those, for whom fitting in with the group is of great importance (see Table 4, column 4). Likewise, respondents from more collectivistic states also reported feeling recognized by others when wearing a mask. This finding highlights how social recognition by others might be important both at the level of the individual as well as the level of one's community; the pattern hints at the possibility that the greater inclination to wear masks among respondents from collectivistic states might be sustained by the approval of other members of the community.

Respondents who reported that their community had a mandatory mask policy also reported feeling greater pride and prestige. It appears that many respondents experienced social recognition when they did comply with official instructions aimed at protecting the community. And although conservatism was otherwise consistently related to more negative feelings about masks, this variable was not correlated with the experience of social recognition.

#### Trust in Public Officials

Different patterns emerged for trust in government officials and trust in public health officials, with the former being substantially lower than the latter (see Table 1). As shown in Table 5, columns 1 and 2, independence and conservatism were both negatively related to trust in government officials, presumably because during a worldwide pandemic, the government might impose restrictions on individual freedoms. Yet, the two interdependence variables predicted higher trust in public health officials but were unrelated to trust in (elected) government officials. Conservatives also trusted public health officials less than liberals, though the coefficient was only half the size of that for trust in government officials.

**Table 5:** Result of multilevel analyses: trust and aversion to masks.

	**Trust Gov't officials**		**Trust public health**		**Negative evaluation**		**Not wanting to be seen with a mask**	
	***b***	**(*se*)**	***b***	**(*se*)**	***b***	**(*se*)**	***b***	**(*se*)**
Intercept	3.20^***^	(*0.31*)	4.01^***^	(*0.23*)	2.68^***^	(*0.27*)	0.87^**^	(*0.28*)
**Individual-level**								
Female (Male = 0)	< -0.01	(*0.11*)	−0.23^**^	(*0.08*)	0.07	(*0.10*)	0.07	(*0.10*)
Education (High School = 0)								
Some college	−0.16	(*0.23*)	0.15	(*0.17*)	−0.37^+^	(*0.20*)	0.21	(*0.21*)
College	−0.24	(*0.21*)	0.27	(*0.16*)	−0.02	(*0.18*)	0.89^***^	(*0.20*)
Advanced Deg.	−0.34	(*0.24*)	0.18	(*0.18*)	−0.04	(*0.21*)	0.82^***^	(*0.22*)
Age (18–24 years = 0)								
25–34	0.11	(*0.23*)	−0.42^*^	(*0.17*)	−0.15	(*0.20*)	0.25	(*0.21*)
35–44	−0.08	(*0.24*)	−0.41^*^	(*0.18*)	−0.10	(*0.21*)	0.11	(*0.22*)
45–54	0.05	(*0.26*)	−0.33^+^	(*0.19*)	−0.01	(*0.23*)	0.09	(*0.24*)
55–64	−0.30	(*0.31*)	−0.26	(*0.23*)	0.19	(*0.26*)	0.65^*^	(*0.28*)
65–74	0.04	(*0.46*)	−0.39	(*0.34*)	−0.09	(*0.39*)	0.03	(*0.42*)
Race/Ethnicity (White = 0)								
Asian	−0.11	(*0.25*)	0.05	(*0.19*)	0.19	(*0.22*)	−0.05	(*0.23*)
Black	−0.15	(*0.18*)	0.13	(*0.13*)	<0.01	(*0.15*)	0.19	(*0.16*)
Latinx	−0.13	(*0.26*)	−0.25	(*0.20*)	−0.15	(*0.23*)	−0.10	(*0.24*)
Other	−0.38	(*0.60*)	0.18	(*0.46*)	0.30	(*0.52*)	−0.54	(*0.55*)
Independence	–0.24^*^	**(*****0.10*****)**	−0.11	(*0.07*)	0.20^*^	**(*****0.09*****)**	−0.15	(*0.09*)
Collective interdep.	0.16^+^	(*0.10*)	0.57^***^	**(*****0.07*****)**	**–**0.04	(*0.08*)	0.13	(*0.09*)
Relational interdep.	0.01	(*0.09*)	0.19^**^	**(*****0.07*****)**	**–**0.03	(*0.08*)	−0.06	(*0.09*)
Conservatism	−0.35^*^	**(*****0.04*****)**	−0.16^***^	**(*****0.03*****)**	0.40^***^	**(*****0.04***)	0.49^***^	**(*****0.04*****)**
Mask Mandatory	−0.04	(*0.14*)	0.16	(*0.11*)	−0.21	(*0.12*)	0.66^***^	**(*****0.13*****)**
**State-level**								
Honor State (No = 0)	−0.130	(*0.126*)	−0.048	(*0.092*)	0.012	(*0.107*)	0.251^*^	**(*****0.119*****)**
Tightness	0.015^*^	**(*****0.006*****)**	−0.001	(*0.004*)	−0.009^+^	(*0.005*)	–0.013^*^	**(*****0.005*****)**
Collectivism	0.005	(*0.006*)	<0.001	(*0.005*)	−0.005	(*0.006*)	0.001	(*0.006*)
GSP	−0.001	(*0.006*)	−0.001	(*0.005*)	0.005	(*0.006*)	<0.001	(*0.006*)
Gini	−0.019	(*0.035*)	−0.006	(*0.026*)	−0.027	(*0.030*)	−0.005	(*0.033*)
**Variance components**								
State		<0.01		0.00		0.00		<0.01
Residual		1.70		0.95		1.27		1.44
**Model fit**								
AIC		2135.01		1795.29		1957.72		2042.61
BIC		2249.50		1909.82		2072.13		2157.14
−2 Log Likelihood		2083.10		1743.29		1905.72		1990.32
ICC		0.03		0.00		0.03		0.05
Marginal *R*^2^		0.14		0.24		0.23		0.38
Conditional *R*^2^		0.15		0.24		0.23		0.38
*N*		596		605		602		605

* *p < 0.05;*

** *p < 0.01;*

*** *p < 0.001*.

*For our main predictor variables, coefficients and their standard errors are bolded, if they are significant at a minimum of p < 0.05*.

Intriguingly, respondents from tighter U.S. states expressed a higher level of trust in government officials. Harrington and Gelfand (2014) demonstrated that state-level tightness was positively correlated with support for greater government restriction in various domains of life. We surmise that governments that impose restrictions, presumably to protect public welfare, are trusted more in tighter state-level cultures.

#### Negative Evaluation

As shown in Table 5, column 3, both respondents high in conservatism and those high in independence seemed to evaluate mask wearing much more negatively than liberals. As observed earlier, independence was also related to a greater intent of wearing a mask in the future. This leads to the conclusion that highly independent people were willing to wear masks *even though* they resented doing so. This pattern, however, is consistent with the notion that independence includes a sense of personal responsibility.

#### Social Image

As summarized in column 4 of Table 5, conservatives were more likely to view masks as a sign of weakness, and as further displayed in column 1 of Table 6, they did not wish to be seen with a mask—consistent with their overall opposition to masks. Individuals with a highly independent self-construal were, however, less likely to agree with the notion that masks indicated weakness, even though they evaluated masks negatively, as discussed above. Whereas others reported that men were more likely to view masks as a sign of weakness (e.g., Capraro and Barcelo, 2020), such a gender effect did not materialize in our data.

**Table 6:** Result of multilevel analyses: weakness, well-being, freedom, and voluntariness.

	**Mask is a sign weakness**		**Low well-being**		**Freedom vs. civic duty**		**Voluntariness**	
	***b***	**(*se*)**	***b***	**(*se*)**	***b***	**(*se*)**	***b***	**(*se*)**
Intercept	1.00^***^	(*0.30*)	1.70^***^	(*0.24*)	1.56^***^	(*0.21*)	2.53^***^	(*0.28*)
**Individual-level**								
Female (Male = 0)	<0.01	(*0.11*)	0.04	(*0.09*)	−0.03	(*0.08*)	−0.07	(*0.10*)
Education (High School = 0)								
Some college	−0.06	(*0.22*)	0.13	(*0.18*)	−0.03	(*0.16*)	0.15	(*0.20*)
College	0.58^**^	(*0.20*)	0.62^***^	(*0.17*)	0.37^*^	(*0.14*)	0.74^***^	(*0.19*)
Advanced Deg.	0.56^*^	(*0.23*)	0.64^***^	(*0.19*)	0.47^**^	(*0.16*)	0.72^***^	(*0.21*)
Age (18–24 years = 0)								
25–34	0.29	(*0.22*)	0.11	(*0.18*)	0.18	(*0.16*)	0.39^*^	(*0.20*)
35–44	0.13	(*0.23*)	−0.04	(*0.19*)	0.13	(*0.16*)	0.38^+^	(*0.21*)
45–54	0.34	(*0.25*)	0.03	(*0.21*)	0.22	(*0.18*)	0.33	(*0.22*)
55–64	0.86^**^	(*0.29*)	0.17	(*0.24*)	0.33	(*0.21*)	0.60^*^	(*0.27*)
65–74	0.01	(*0.44*)	−0.11	(*0.36*)	0.18	(*0.31*)	−0.03	(*0.40*)
Race/Ethnicity (White = 0)								
Asian	−0.19	(*0.24*)	−0.06	(*0.19*)	0.12	(*0.17*)	0.37^+^	(*0.22*)
Black	0.22	(*0.17*)	0.05	(*0.14*)	−0.04	(*0.17*)	0.18	(*0.15*)
Latinx	0.16	(*0.25*)	0.12	(*0.21*)	0.14	(*0.18*)	0.50^*^	(*0.23*)
Other	−0.03	(*0.58*)	0.31	(*0.47*)	−0.28	(*0.41*)	−0.43	(*0.52*)
Independence	–0.21^*^	**(*****0.10*****)**	−0.15^+^	(*0.08*)	−0.03	(*0.07*)	−0.18^*^	**(*****0.09*****)**
Collective interdep.	0.03	(*0.09*)	0.13^+^	(*0.08*)	−0.19^**^	**(*****0.09*****)**	0.29^***^	**(*****0.09*****)**
Relational interdep.	−0.05	(*0.09*)	−0.12	(*0.07*)	**–**0.09	(*0.06*)	0.08	(*0.08*)
Conservatism	0.46^***^	**(*****0.04*****)**	0.41^***^	**(*****0.03*****)**	0.49^***^	**(*****0.03***)	0.18^***^	**(*****0.04*****)**
Mask Mandatory	0.57^***^	**(*****0.14*****)**	0.41^***^	**(*****0.11*****)**	0.26^*^	**(*****0.10*****)**	−0.49^***^	**(*****0.13*****)**
**State-level**								
Honor State (No = 0)	0.239^*^	**(*****0.119*****)**	0.217^*^	**(*****0.010*****)**	0.174^*^	**(*****0.084*****)**	0.161	(*0.142*)
Tightness	−0.017^**^	**(*****0.005*****)**	−0.015^***^	**(*****0.004*****)**	–0.119^**^	**(*****0.039*****)**	< -0.001	(*0.006*)
Collectivism	−0.001	(*0.006*)	−0.002	(*0.005*)	<0.001	(*0.004*)	−0.006	(*0.007*)
GSP	< -0.001	(*0.006*)	– <0.001	(*0.005*)	0.002	(*0.005*)	0.003	(*0.008*)
Gini	0.027	(*0.033*)	−0.017	(*0.027*)	−0.022	(*0.023*)	−0.026	(*0.004*)
**Variance components**								
State		0.00		0.00		0.00		0.06
Residual		1.58		1.04		0.80		1.27
**Model fit**								
AIC		2093.45		1831.16		1690.05		1983.92
BIC		2207.98		1945.44		1804.50		2098.46
−2 Log Likelihood		2041.45		1779.16		1638.05		1931.92
ICC		0.05		0.05		0.04		0.04
Marginal *R*^2^		0.33		0.35		0.41		0.20
Conditional *R*^2^		0.33		0.35		0.41		0.24
*N*		605		599		603		605

+ *p < 0.051*,

* *p < 0.05;*

** *p < 0.01;*

*** *p < 0.001*.

*For our main predictor variables, coefficients and their standard errors are bolded, if they are significant at a minimum of p < 0.05*.

As predicted based on the honor culture framework, regardless of their personal characteristics, respondents from honor states were also more likely to say that they did not want to be seen wearing a mask. They were also more likely to consider a mask as a sign of weakness than was the case for respondents from non-honor states. This pattern is consistent with the observation that people in honor culture emphasized appearing tough and strong (e.g., Üskül et al., 2019)^8^.

Likewise, among respondents from tighter states, the sentiment that masks were a sign of weakness or spoiled one's public image was less likely shared, consistent with the notion that people in such states were more oriented toward complying with social norms. However, there was again some evidence that respondents in jurisdictions with a mask mandate were particularly likely to characterize masks as a sign of weakness and to reject wanting to be seen with masks (Table 5, column 4, and Table 6, column 1, respectively). We speculate that this is evidence of a backlash against government requirements that were perceived as a limitation on personal freedom (e.g., Microsoft News, 2020; Pawlowski, 2020).

#### Well-Being Consequences

As indicated in Table 6, column 2, conservatives were again more likely to attribute negative consequences to wearing a mask in terms of their emotional well-being. Similarly, respondents who indicated that they lived in jurisdictions with a mask mandate also reported lower well-being. Recall that respondents in such jurisdictions were not any more or less likely to wear masks in public than those in jurisdictions without mask mandates (see Table 3, column 1). Hence, it is conceivable that the attribution of masks generating lower well-being is not exclusively borne out of personal experience. Consistent with this theme, being from a tighter state seemed to protect respondents from a negative impact on their well-being, presumably because, as demonstrated above, masks were not evaluated as negatively, and there was less ambiguity concerning the social meaning of mask-wearing.

Likewise, respondents from honor states said that wearing a mask decreased their well-being compared to those from non-honor states. Though not anticipated, this finding is consistent with honor culture insofar as individuals may experience wearing a mask as a loss of honor, because it conveys weakness rather than personal strength. This, in turn, lowers their well-being and makes, them feel looked down upon by their community (Brown, 2016). Again, there is no evidence that respondents from honor and non-honor states differed in their frequency of mask wearing (see Table 3, column 1). Therefore, it is plausible to assume that respondents responded based on their cultural understanding of what wearing a mask signifies.

#### Freedom, Duty, and Voluntariness

Our analysis of whether respondents perceived mask wearing as an infringement of their freedoms or an act of civic duty revealed that, as expected, conservatives preferred the former interpretation, whereas liberals preferred the latter. Consistent with the notion that government action may arouse a reactance-like response or a backlash, respondents in jurisdictions with a mask mandate considered the mandate an infringement on their freedom (Table 6, column 3). Yet, those with high levels of collective interdependence were more likely to view mask wearing as an act of civic duty. This was also the case for those from tighter U.S. states, where compliance with social norms and rules is valued and enforced. Respondents from honor states, however, were more likely to express that masks wearing was an infringement on their freedoms than was the case for respondents from non-honor states.

If they resided in a jurisdiction with the mask mandate, respondents did not believe that it was individuals' personal decision whether to wear a mask or not. Likewise, those high in independence, who presumably champion personal autonomy, considered mask wearing not a voluntary matter. Yet, respondents who scored high in collective interdependence did feel that there was little social pressure and that individuals made personal and voluntary decisions to wear a mask. At first blush, this might seem surprising in light that this group of individuals also agreed that wearing a mask represented a social norm (see Table 4, column 3). The key to understanding this positive coefficient might be the insight that for those high in interdependence, an external obligation does not have to be experienced as a limitation on their own actions. Rather, it might be experienced as individuals wanting to engage in behavior for the benefit of others [see also Berg et al. (2001), e.g., Janoff-Bulman and Leggatt (2002)].

Unexpectedly, conservatism was also related to the perception of higher levels of voluntariness. Whereas conservatives had a much less favorable view of masks and mask wearing than was the case for liberals, they were also less likely to wear masks regularly (see Table 3, column 1). The greater perceived voluntariness of mask wearing may be because conservatives rejecting the social expectation that otherwise seems to have produced very high levels of mask wearing; instead, they might assert their own agency in deciding when (and when not) they are willing to wear masks. By contrast, liberals might be wearing masks habitually; hence, for liberals mask wearing may simply not be the subject to any reasoned voluntary decision-making process.

### Mediation Analyses

#### Individual-Level Mediation

Given that all of our individual-level cultural variables were involved in predicting aspects of mask-wearing behavior, we tested which specific responses to masks mediated these effects. We performed a series of three mediation analyses in which we predicted past mask-wearing behavior, future intent, and willingness to change one's behavior based on conservatism, collective interdependence, relational interdependence, and independence. As simultaneous mediators, we explored knowledge; perceived utility; providing feelings of protection; whether mask wearing represented a social norm; the experience of social recognition; negative evaluations; whether masks are a sign of weakness; whether participants did not want to give others the satisfaction of seeing them with a mask; low well-being; and whether wearing mask represented an infringement of freedom or a civic duty (total of 10 mediators)^9^^,^^10^. We examined potential mediational relationships among individual-level variables in the context of our multilevel design. All multilevel mediation analyses were carried out in Stata 14.2 using the *gsem* function. In all three models we controlled for gender, education, age, and race.

We aimed to generate specific hypotheses for conservatism as to which mediator might be most critical. However, we expected (and found) that conservatives evaluated all aspects of masks more negatively than liberals; hence, for conservatism, we used mediation analysis to explore the most potent mediators. For collective interdependence, we expected that those high on this dimension would consider it much more normative, and in the interest of their community to wear masks. Likewise, we suspected that people high in collective interdependence would wear masks to the extent that they receive some social recognition from wearing a mask.

Pertaining to relational interdependence, we suspected that one's desire for others to feel protected would serve as a mediator on mask-wearing behavior. Lastly, we hypothesized that the effects of independence on mask-wearing behavior would be mediated by the perception of the utility of doing so, and that it would be a civic duty to wear a mask (see Supplementary Material S8 for additional details).

**Figure 1:**
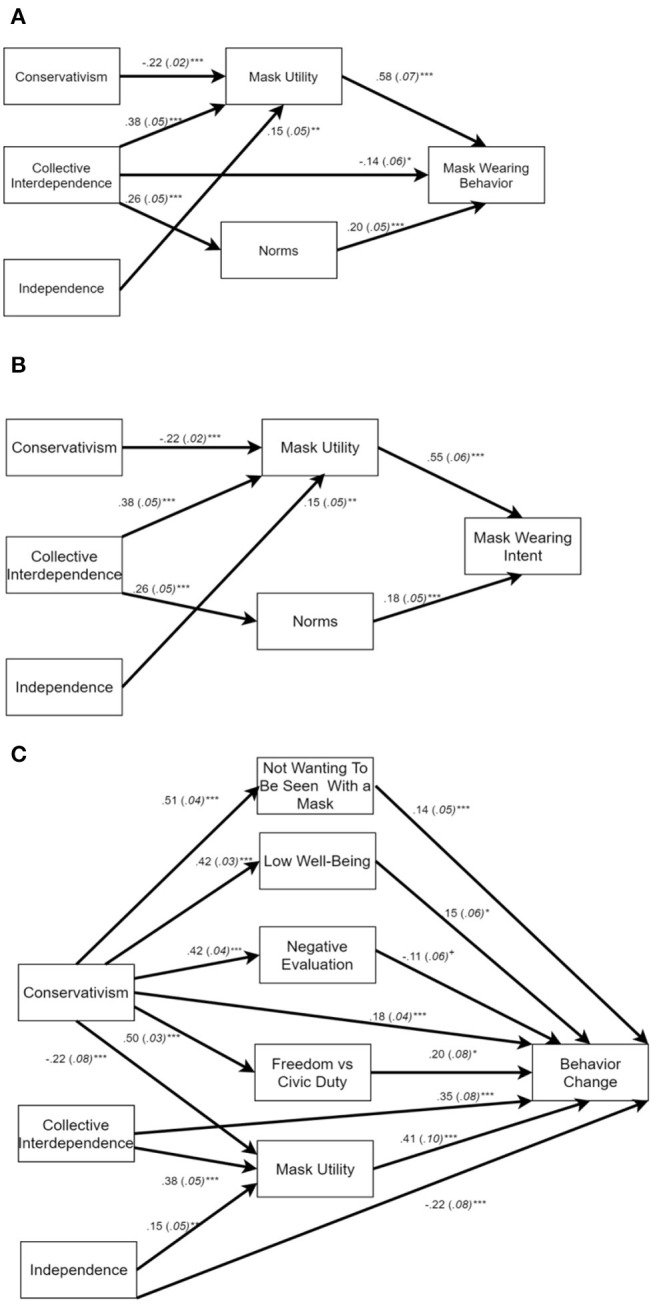
Mediation models pertaining to mask-wearing behaviors. The figures depict only paths involved in a reliable indirect (mediation) effect; and significant direct effects of the cultural predictors onto the behavior variables and onto the mediators. **(A)** refers to past mask wearing behavior; **(B)** to future mask wearing intent; and **(C)** to willingness to change one's behavior. *p < 0.05; **p < 0.01; ***p < 0.001.

Below we summarize our mediation results focusing on statistically reliable indirect effects, which are displayed in Figure 1. Because our interest is on the implications of our cultural predictors, we discuss findings separately for conservatism, the two interdependence variables, and independence.

##### Conservatism

Our models revealed that the statistical effects of conservatism onto past behavior and onto future intent to wear a mask was exclusively mediated by the perceived utility of wearing a mask, indirect effects *ab* = −0.130, 95% *CI* [−0.169, −0.090] and *ab* = −0.122, 95% *CI* [−0.158, −0.086], respectively. Higher levels of conservatism were related to lower perceived utility, which in turn predict more frequent past and future mask wearing (see Figures 1A,B). The same mediational relationship was also present for willingness to change behavior, *ab* = −0.079, 95% *CI* [−0.122, −0.037]; yet, conservatives were also willing to change their behavior to the extent that they did not want others to see them with a mask, *ab* = −0.075, 95% *CI* [0.022, 0.128]; to the extent that they reported low well-being as a result of wearing a mask, *ab* = 0.068, 95% *CI* [0.013, 0.122]; and to the extent that they considered mask wearing as an infringement on their freedom, *ab* = 0.096, 95% *CI* [0.014, 0.178] (see Figure 1C). As established above, among conservatives there was a willingness to reduce the frequency of mask wearing, never a willingness to increase one's frequency of mask wearing. In short, conservatives seem to have a variety of reasons at the ready for why they might no longer wear masks. Yet, in the immediate conservatives' lower self-reported mask-wearing behavior seemed to be primarily predicted by them considering mask wearing as not useful.

##### Collective Interdependence

As anticipated, whether mask wearing was considered a social norm served as a mediator for past behavior and future intent of wearing a mask, indirect effects *ab* = 0.053, 95% *CI* [0.018, 0.088] and *ab* = 0.047, 95% *CI* [0.017, 0.078], respectively. Confirming our hypothesis, those high in collective interdependence considered it a social norm to wear masks, which predicted more frequent mask wearing in the past and greater intent to wear masks in the future. A parallel mediational path was found for perceived utility, with those high in collective interdependence considering it simply more useful, and in everybody's interest, to wear masks, indirect effects *ab* = 0.224, 95% *CI* [0.146, 0.301] and *ab* = 0.211, 95% *CI* [0.140, 0.281], respectively. Collective interdependence was also related to greater willingness to change one's behavior, to the extent that they considered the perceived utility of mask wearing to be high, indirect effect *ab* = 0.157, 95% *CI* [0.074, 0.240]^11^.

Overall, this analysis partially confirmed our expectations, though we did not find any evidence that social recognition derived from wearing a mask served as a mediator.

##### Relational Interdependence

With none of the mediational relationships being reliable, our hypothesis concerning to this variable was not confirmed.

##### Independence

As predicted, independence predicted past masking wearing behavior and future intent to the extent that those high in independence perceived the utility of mask wearing to be high, indirect effects *ab* = 0.224, 95% *CI* [0.146, 0.301] and *ab* = 0.211, 95% *CI* [0.140, 0.281]. The same type of mediational relationship was also reliable with regard to willingness to change one's behavior, indirect effect *ab* = 0.062, 95% *CI* [0.012, 0.111]. Whereas this confirmed that independents would be responsive to the utility of mask wearing, there was no evidence to confirm that any effects of independence were mediated by viewing mask wearing as a civic duty.

#### State-Level Mediation

**Figure 2:**
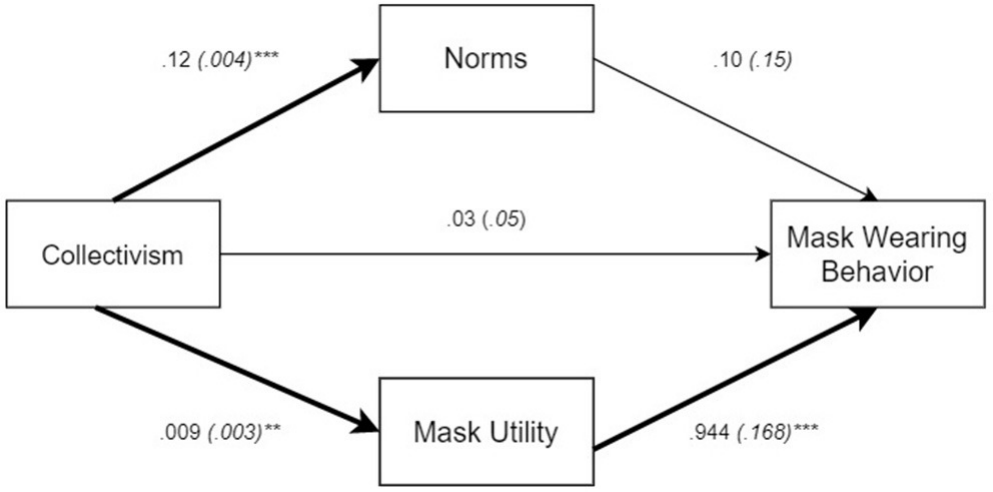
Mediation model pertaining to past mask wearing at the state-level. **p < 0.01; ***p < 0.001.

To examine whether this critical mediational analysis would generalize to the societal level, we examined whether the relationship between state difference in collectivism and past mask wearing was mediated by perceived utility and social norms. For this analysis, we aggregated the individual-level mediators as well as past mask wearing and performed a mediation analysis with states (*k* = 45) as units of analysis. The analysis was carried out in R's *lavaan* (Rosseel, 2012). As summarized in Figure 2, state-level collectivism predicted both state differences in average social norms and perceived utility; however, only state-averages in perceived utility emerged as a significant mediator, indirect effect *ab* = 0.009, 95% *CI* [0.001, 0.017], but not for state-averages in the extent to which wearing masks was considered a social norm, indirect effect *ab* = 0.001, 95% *CI* [−0.001, 0.003]. Additional analyses in which we used state averages of making others feel protected and social recognition and as mediators only confirmed that only perceived utility resulted in a reliable indirect effect (see Supplementary Material S9).

## Discussion

In the midst of the worsening COVID-19 crisis of 2020, the use of masks in the U.S. was plagued by deep cultural and political divisions. The goal of this study was to examine mask-wearing behavior and the cultural understandings of masks within the context of four broad frameworks within cultural psychology, namely, research in individualism-collectivism, tightness-looseness, honor culture, and political orientation (conservatism/liberalism).

Overall, political orientation was the most pervasive predictor (see also Blakemore, 2020). Individuals who described themselves as conservative were less likely to believe that wearing masks generates benefits and thus were less likely to wear masks—a finding suggested by our mediation analyses. Conservatives also expressed that masks undermine their well-being and public image. As cultural symbols, for conservatives masks represented weakness and a limitation on their individual freedom. These findings are certainly no surprise in the context of the American political landscape of 2020, an election year, which was characterized by deep divisions among the U.S. electorate. Our data provide a glimpse of the depth and intransigence of this divide: conservatives, more so than liberals, indicated a willingness to change their mask-wearing behavior if their community and those close to them expected them to do so. Yet, as we determined, conservatives were willing to *reduce* their mask-wearing behavior; they did not express any willingness to wear masks more frequently. It is difficult to know if conservatives around the U.S. did maintain a lower level of mask wearing during the last 5 months of 2020, that is, following the completion of our study. However, it is almost certain that greater adherence to non-pharmaceutical public health measures, such as wearing masks, would have prevented the explosion of new COVID-19 cases in the U.S. as it occurred between October 2020 and January 2021 (cf. Li et al., 2020b; Singh et al., 2021).

The second most pervasive predictor in our study was individual differences in interdependent self-construals as they relate to collectives. Respondents high on this dimension had not worn masks more frequently, nor did they know more about the virus than others. Rather, they considered wearing masks to be a normative behavior, and not only considered it a civic duty but also experienced mask wearing as socially rewarding. As suggested by a mediation analysis, both the perceived utility of wearing masks and the perceived normativeness of the behavior seemed to motivate them to wear masks more frequently.

For relational interdependence we obtained similar results as for collective interdependence; however, this variable was unrelated to greater perceived utility, nor was it related to a conception of mask wearing as civic behavior–consistent with implied focus on interpersonal concerns. We did confirm our expectation that relational interdependence was related to a desire to make others feel protected, though this demonstrated relationship emerged for either interdependence dimension. Relational interdependence did predict a higher level of knowledge about COVID-19, though this greater knowledge did not have any implications for behavior.

Independent self-construals emerged as yet another important predictor. Those high on this dimension expressed a greater willingness to wear masks in the future and perceived the utility of masking to be high. But even though they also viewed wearing masks to be normative, individuals high in independence resented masks more than their low-independence counterparts. They also indicated that they saw mask wearing to be the result of social pressure, rather than the result of individuals' voluntary decisions. The fact that independents distinguish between cooperation with a community demand and their personal evaluation of and desire to wear masks points to a substantially different process. Collective interdependence seemed to entail that individuals embrace their community's norms and requirements as a personal goal (cf. Janoff-Bulman and Leggatt, 2002). By contrast, the pattern observed for independence seems to be consistent with individuals assuming responsibility; even though a particular action, such as wearing masks, is perceived to be personally unpleasant, nevertheless individuals carry it because it is deemed beneficial (Waterman, 1981, 1984).

Beyond individual-level differences in independence and interdependence, our work also revealed several state differences for collectivism. Recall that Vandello and Cohen's (1999) measure of collectivism focuses primarily on culturally relevant behaviors (e.g., divorces and carpooling) and residential structure (e.g., living arrangement) rather than self-reported value preferences. Not only were respondents from collectivistic states more likely to report having worn masks, but respondents from such states also perceived greater utility in mask wearing, they considered it more normative, were more likely to derive social recognition from wearing masks, and were more likely to agree that masks may make other people feel protected. Though our state-level mediation analysis suggested that the mask-wearing behavior of the residents of collectivistic states may have been mainly the result of greater perceived utility, the nature of our findings make clear that there are culturally shared perceptions that existed in different states. Regardless of whether they endorsed higher or lower levels of collective interdependence, people in collectivistic states agreed that wearing masks was beneficial, which seemed to motivate them to wear masks more frequently.

Tightness-looseness also emerged as a cultural predictor. Respondents from tighter states were more likely to trust government officials, and less likely to consider masks to symbolize weakness, but instead they regarded wearing masks as a civic duty, rather than an infringement of freedom—in addition to being less likely to attribute negative well-being consequences to masks. Presumably aided by the perceptions of masks as an official requirement issued by a trusted source (government), respondents from tighter states were less willing to change their mask-wearing behavior. The present study did not unveil any evidence that residents from tighter states wore masks more frequently than those from looser states, as we predicted based on Gelfand et al. (2021). Recall that these authors argued that tighter societies were more successful at fighting the COVID-19 pandemic because people were more likely to wear face masks. Still, our data nevertheless showed that cultural tightness seemed to promote an atmosphere supportive of wearing masks. It is likely that in culturally tight states there may have existed less ambiguity as to what the rules were (e.g., Gelfand et al., 2011; Harrington and Gelfand, 2014). Our results are consistent with the notion that mask-wearing behavior is inherently embedded within the social norms of a community. At the most general level, our findings affirm the relevance of cultural differences of tightness-looseness in the pandemic, which has been shown to predict COVID-19 cases and deaths on a global scale (Gelfand et al., 2021) as well as population responses to the pandemic (Cao et al., 2020; Im and Chen, 2020).

Finally, as predicted, honor culture was related to the belief that masks were a sign of weakness. Because projecting personal strength is prized, respondents from honor states also said that they did not want to give others the satisfaction of being seen with a mask—reminiscent of the May 2020 quote by Donald Trump (Carlisle, 2020). Consistent with recent theorizing by Brown (2016), respondents from honor states were also more likely to say that masks reduced their well-being than respondents from non-honor states. Although some scholars have argued that honor culture is fading in the U.S. (Grosjean, 2014), our study showed that the psychological concerns of honor cultures persist and that they are being applied to assign social meaning to masks and mask wearing.

An important takeaway from this research is that masks are not simply a useful tool in shielding oneself from infection; they are also symbols, which take on different meanings across political and cultural contexts. Our data demonstrated that individuals view masks through the lens of existing cultural (and political) beliefs. As a result of the application of these beliefs, individuals attribute value and utility to masks and mask wearing. Whereas this was most explicit at the individual level, the same general process also seems to occur at the societal level. Collectivistic states seem to embrace mask wearing as a useful, socially normative behavior, that is rewarding to the mask wearer, and which is reassuring to members of the community. Tighter states appear to treat masks as a legitimate requirement by the authorities which enhances the favorability of their evaluation and makes people unwilling to change their mask-wearing behavior. Honor states seem to treat masks as a challenge to one's public image, which has the potential of undermining well-being^12^.

Even though our findings confirmed many of our predictions, critics might wonder to what extent cultural concepts are relevant to public health. This question is inherently tied to cultural variables being able to account for actual behavior.

We identified two variables that predicted past behavior directly: Conservatism, of our four individual differences in cultural beliefs, as well as collectivism, one of our three dimensions of between-state differences. Two additional cultural variables emerged as predictors of past behavior in our mediation analyses, namely, collective interdependence, and independence^13^. From this perspective, the present research yielded an array of cultural variables that predict mask-wearing behavior. In other words, different aspects of culture appear to be involved in whether individuals don masks or not. Strikingly, even cultural constructs that are unrelated (if not conceptually opposed), such as independence and collective interdependence appear to predict higher levels of mask wearing. Perhaps even more surprisingly, conservatism and interdependence tend to be substantially correlated; yet, conservatism predicted a great reluctance to wear masks, whereas interdependence predicted a greater readiness to wear masks. How is this possible?

Our mediation analyses produced suggestive evidence that points to a single process: different cultural dispositions seem to increase (or decrease) mask behavior to the extent that individuals perceive mask wearing to possess greater (lower) utility. That is, conservative respondents perceived mask wearing to less useful, and in the interest of themselves and others than was the case for liberal respondents. Likewise, respondents high in collective interdependence were more likely to consider masks useful than respondents low on this dimension, and a very similar pattern emerged for respondents high and low in independence. We made the very same observation at the state level, such that high-collectivism states predicted higher state averages of perceived utility, which then translated into higher mask wearing at the state level. In short, cultural dispositions, both at the individual level as well as at the state level seem to orient individuals' perspectives toward an evaluation of what is useful and in their interest. This process is reminiscent of the unitary process of utility maximization that is at the heart of rational choice theory: whatever considerations are entertained when making a decision, ultimately individuals will choose to engage in actions that they consider most useful in advancing their interests (e.g., Scott, 2000).

If perceived utility is the critical ingredient to motivate mask wearing, then researchers must exercise great care when they are tempted to condemn the fact that individuals refuse to wear masks, even when scientific literature supports that masks will curb infection (e.g., Singh et al., 2021). The reluctance of conservatives to wear masks should not necessarily be interpreted as irrational, or as obstinate resistance to engaging in a salutary behavior. Rather, if conservatives do not perceive utility in wearing masks, this implies that, at least from their own perspective, they are acting in the best interest of themselves and others. Yet, objective reality may have the potential of correcting these beliefs. During the pandemic, and all else being equal, communities in which mask wearing is rare face a much higher risk of infection than communities in which mask wearing is common. If individuals' perceptions of mask wearing utility are tethered to real-world outcomes, then one may be confident that, at least over time, individuals who dismiss the utility of wearing masks may come around, and rationally adjust their beliefs. Hence, as the pandemic may have dragged on, there is a good chance that at least some conservatives will have changed their mind.

Whereas, this may be a reason for optimism, it is easy to see that any process of belief change may take time, and be of little comfort when a surging wave of infection demands immediate action. Moreover, social judgment research has yielded much evidence of individuals engaging in motivated reasoning, allowing them to focus on observations that are consistent with prior beliefs, use information creatively in support of preference conclusions, and ignore facts that are inconsistent with expectations (e.g., Kunda, 1990). Still, over the long-term, our analyses suggest that, especially as the pandemic worsened significantly in the months following the completion of our survey, individuals will have adjusted their beliefs about the utility of masks.

Beyond utility, the only other mediator was whether respondents perceived mask wearing to be socially normative or not. This variable helped explain the effects of collective interdependence on the future intent to wear masks. This finding is consistent with the notion that those who feel committed to their group are also likely to adhere to its norms and rules. The fact that this pattern only emerged for behavioral intent, and not for past behavior raises questions. One wonders whether individuals high in collective interdependence merely affirmed their membership in the group by expressing this intent, even when the actual execution of the behavior may not have been consistent with the intention.

### Implications

Our research has established that masks and mask wearing are deeply embedded in their cultural context, with their symbolic significance being critical to people [see also Sunstein (2020) and Timpka and Nyce (2021)]. Any future attempt aimed at increasing mask-wearing behavior, whether during the present COVID-19 pandemic or a future epidemic, should take into consideration the cultural meanings of masks—and any public health measure that the population is unaccustomed to. For instance, when directed at honor cultures, public health communication might seek to frame masks as a symbol of unity and strength. Indeed, many individuals used masks not only as a quasi-fashion accessory, but also as a canvas, e.g., to display the flag, or communicate various messages. By the same token, to appeal to an audience that is likely to view any requirement to wear masks with suspicion, it is critical to seek out an avenue of communication to avoid cultural “red flags.”

### Limitations

As with any study, our research faced a number of limitations. First, our first limitation refers to the fact that we only assessed self-reports of mask-wearing behavior. It is an open question to what extent self-reported mask-wearing corresponds to objective behavior. Even if we know of no study that has yet examined the accuracy of self-reports, a German study conducted early in the pandemic suggested an over-reporting bias (Kovacs et al., 2020). On the one hand, it is easy to see that respondents would overreport a behavior that was considered desirable during a pandemic, and we cannot exclude the possibility that this also occurred in our data. On the other hand, especially respondents who described themselves as conservative reported wearing masks less frequently than liberals [see also Blakemore (2020)]. To the extent that a self-reporting bias existed, it did not erase this expected difference between respondents with different political leanings^14^.

Second, we concede the possibility that our research may have been unable to detect some existing relationships because of a heavy skew in respondents' self-reported behavior. The overwhelming majority of our sample indicated having worn a mask *always* or *most of the time*. Whereas this is good news from the perspective of public health, such an apparent ceiling effect may have constrained variability and made it statistically difficult to identify relationships between variables that may have been present in our data.

Third, because we did not sample all 50 states our data do not allow inferences about the cultural patterns of Alaska, Delaware, Vermont, or the Dakotas. By the same token, with per-state sample sizes being highly variable, a potential weakness is that we did not capture the prevalent views of different state populations equally well. Moreover, future research may need to affirm the present findings as our study did rely on Mturk workers, whose characteristics did not match the general population. Even though there is no reason to believe that Mturk workers self-selected differently across states, future research might need to corroborate this assumption.

But in spite of these limitations, we believe that our study does shed light on how cultural and political patterns in the U.S. help shape behavior and response related to masking, one of the most critical tools in fighting airborne pathogens.

## Conclusion

In closing, for most of 2020, the U.S. saw its global leadership role challenged by its poor handling of the COVID-19 pandemic. As of this writing (May 2021), the vaccination campaign in the U.S. is one of the most successful in the world, especially considering the size of the U.S. population. However, believing the pandemic to be effectively over, many individuals are less likely to wear masks than only months ago. Many U.S. states, eager to reinvigorate their economies, have dropped their mask mandates (e.g., Texas and Louisiana). The consequence is either a decline of new infections that is lower than desirable or a resurgence of cases, likely aided by newer and faster-spreading variants of the SARS-Cov-2. Because masks, along with other non-pharmaceutical interventions, are still among the easiest and most effective ways of limiting the spread of infection, the question of how Americans relate to and attribute meaning to masks remains relevant. Indeed, the hope that the insight gained about human behavior during the COVID-19 pandemic will help prepare the world for the next pandemic–whenever it may occur.

## Data Availability Statement

The datasets generated for this study can be found in online repositories. The names of the repository/repositories and accession number(s) can be found at: https://www.researchgate.net/profile/Markus_Kemmelmeier/publications or https://www.researchgate.net/profile/Waleed_Jami2.

## Ethics Statement

The studies involving human participants were reviewed and approved by Institutional Review Board, Office of Research Integrity, University of Nevada, Reno, IRBNet ID: 1629853-1. The patients/participants provided their written informed consent to participate in this study.

## Author Contributions

MK conceived the project, the design, and generated the materials. WJ assisted with the materials and implemented the materials in Qualtrics. All authors collaborated in collecting the data, cleaning, data analysis, and contributed to the writing of the paper.

## Conflict of Interest

The authors declare that the research was conducted in the absence of any commercial or financial relationships that could be construed as a potential conflict of interest.

## Footnotes

^1^Our discussion of honor culture focuses exclusively on honor cultures with an independent cultural mandate, as it exists in the United States (e.g., Nisbett and Cohen, 1996; Brown, 2016), or used to exist in parts of the British Isles (e.g., Richerson and Boyd, 2005). For a more comprehensive review, and consideration of different kinds of honor cultures, see Üskül et al. (2019).

^2^Treating liberalism-conservatism as a cultural distinction does not deny the role of genetics (Hibbing et al., 2013) or personality differences (Carney et al., 2008) as predictors of liberal or conservative attitudes.

^3^The items left it open whether respondents would increase or decrease their use of masks. Those reporting that they wear masks regularly could decrease their use of masks; people who do not wear masks might increase their use of masks.

^4^Separating the first four items, and the second three into two separate indices produced parallel results throughout the paper, including mediation analyses. Hence, they were collapsed.

^5^The second item was inspired by President Trump's comments from May 21, 2020, about not wanting to give journalists the satisfaction of seeing him wearing a mask.

^6^Self-reports are contingent on the response scales being offered (e.g., Schwarz, 1999). Blakemore (2020) only offered four response options (shares of responses in parentheses): *Always* (60%), *Sometimes* (25%), *Rarely* (7%), and *Never* (8). Focusing on the 60% *Always* responses in Blakemore's data, some commentators (Timpka and Nyce, 2021) bemoaned the low levels of mask wearing. This approach might mischaracterize the actual frequency. Note that our survey offered two additional response options between *Always* and *Sometimes*: *About half the time* and *Most of the time*. We surmise that with 91% of our respondents indicating a frequency of *About half the time* and higher, and only 6% of our respondents choosing the *Sometimes* option; our approach may have been better equipped to capture mask wearing that occurred frequently but not necessarily consistently.

^7^Though not included in Table 2B, the present data showed that honor states did have a lower share of Democratic legislators (*r* = −0.30), reminiscent of Harrington and Gelfand's (2014) finding that honor states include a higher proportion of Republican voters.

^8^Supplementary analyses did not reveal any gender effect that might have occurred differentially in honor and non-honor states.

^9^Testing multiple simultaneous mediational relationships implied that we would be able to control the effects of overlapping mediators, with indirect effects only reflecting the unique contribution to a particular mediator.

^10^As before, in the mediation analysis for willingness to change behavior we controlled for participants' past behavior.

^11^Follow-up analysis revealed that this mediational relationship was strongest among those individuals who did not wear masks all the time or most of the time. It was very weak to nonexistent among participants who said that they wear a mask all the time. Hence, among individuals high in collective interdependence, greater perceived utility of masks seems to imply a willingness to *increase* the frequency of mask wearing.

^12^A state-level analysis, which examined whether interpreting masks as a sign of weakness mediated the link between honor status and state-level well-being revealed a significant indirect effect, ab = 0.208, 95% [0.024, 0.392]. Respondents from honor states were more likely to think that masks were a sign of weakness; agreeing with this sentiment lowered respondents' well-being.

^13^Mediation analysis does not require there to be a significant relationship between a predictor and an outcome variable in order for the same predictor to exert a statistical influence on the outcome variable via a mediator. This is the result of tests of mediational relationships having more statistical power than those pertaining to direct relationships (Kenny and Judd, 2014).

^14^A threat to the validity of our findings stems from the possibility that self-reports of behavior were entirely driven by differential responses biases, such that conservatives feel compelled to self-report infrequent mask wearing (regardless of their actual behavior), whereas liberals feel compelled to report frequent mask-wearing behavior (again, regardless of their actual behavior). We do not consider this plausible because our self-reports of mask-wearing behavior do replicate, albeit weakly, the same gender differences obtained in an observational study of mask-wearing behavior (Haischer et al., 2020). The self-report study by Blakemore (2020) also obtain patterns similar to those reported by Haischer et al. (2020). Even when self-reports are subject to potential distortion, they do track objectively assessed behaviors.

## Supplementary Material

Details concerning sampling, analytical approach, and results are provided in the Supplementary Materials. Section numbers in the text refer to corresponding section numbers in the Supplementary Materials.
