# Supplementary material for: Mask Wearing as Cultural Behavior: An Investigation Across 45 U.S. States During the COVID-19 Pandemic
Source: Front Psychol. 2021 Jul 21;12:648692. doi: 10.3389/fpsyg.2021.648692 (PMC8333278; doi:10.3389/fpsyg.2021.648692)
Supplement: Supplementary file 1 [file Data_Sheet_1.docx]

Mask wearing as cultural behavior: An investigation across 45 U.S. states during the COVID-19 pandemic

Markus Kemmelmeier^1*^, Waleed A. Jami^1^

^1^Interdisciplinary Social Psychology Ph.D. Program, University of Nevada, Reno, Nevada, USA

***** Corresponding author: markusk@unr.edu

Supplementary Materials

Contents

**S1 Supplementary Information pertaining to Respondents**

**S2 Supplementary Information pertaining to Dependent variables and Self-construal**

**S3 Supplementary Information pertaining to Cultural differences between U.S. states**

**S4 Supplementary Information pertaining to State-level Control Variables**

**S5 Supplementary materials pertaining to Individual-level predictors**

**S6 Supplementary Information pertaining to Analytical Approach**

**S7 Supplementary Analyses pertaining to Perceived Utility**

**Table S7.1**

**Table S7.2**

**S8 Individual-level mediation**

**S9 State-level mediation**

**References**

**S1 Supplementary Information pertaining to Respondents**

**Power Considerations**

The determination of sample size requirements in the case of multilevel designs is complex (e.g. Snijders, 2005). The goal was to test fixed effects at level-1 (individual respondents) and level-2 (U.S. states), but not for cross-level interactions, though the primary consideration in the determination of these minimal criteria was the detection of between-state differences. Whereas the chances of detecting such level-2 differences increases with the number of states included in the sample, in the case of the U.S., there is a natural upper limit of 50. Likewise, an increase of the number of respondents per state (i.e. number of level-1 observation) would primarily affect the examination of level-1 differences. Though no data were available to support this, we assumed the intraclass correlations would be modest, with any design effects being small.

The goal of the present study was to recruit a sample of at least 600 respondents from all of the 50 U.S. states (i.e. 12 respondents per state). However, because we expected considerable variation in the participation from different U.S. states, there was the additional stipulation that none of the state-level samples would be smaller than *n* = 6. Based on Scherbaum and Ferreter (2009), a multilevel design with 40 units and 6 observations per unit yielded a statistical power of about .80 concerning the detection of a midsize between-state (level-2) effect, assuming an alpha of .05 (see their Figure 1). Moreover, the same authors reported a statistical power of .88 for 12 observations. Focusing on level-1 analyses, if we were to assume no between state differences, with an alpha of .05 and a beta of .80, a sample of 600 observations provided sufficient power to detect a total of only 3.7% of variability in the dependent variable (assuming the 23 predictors deployed in Tables 3 through 7). With the expected design effect being very small (< .10), but with variances of the predictors and the dependent variables unknown, a sample of 600 respondents was being deemed large enough to detect even modest level-1 effects.

**Recruitment and Sample**

Using Amazon’s Mechanical Turk service (Mturk), we recruited a total of 841 respondents, who were directed to our online survey on Qualtrics®. Our instructions emphasized that only U.S. residents who were currently located in one of the 50 states would be eligible for participation. The study was expected to take between 9 and 12 minutes, and respondents received $1 in exchange for their participation. Data collection took place between July 20 and August 2, 2020. We retained a total of 633 respondents because 208 respondents had to be removed. We detail the exclusion criteria below, as well as how many respondents were removed for which reason.

**Removal of untoward cases**. Of the 208 removed cases, a total of 43 respondents accessed the study, but did not answer any questions. Because it was critical to ascertain that respondents were residents of a U.S. state, we excluded all respondents whose IP address/GPS information located them in a country other than the U.S. (see Bai, 2018).^[[1]](#footnote-1)^ We also excluded all respondents who indicated in their open-ended responses that they were not currently located in the U.S. This eliminated another 24 respondents.

Because of the recent “Mturk quality crisis” and in line with recent recommendations (e.g., Chmielewski & Kucker, 2020; Kennedy et al., 2020), we applied stringent exclusion criteria concerning data quality. We eliminated 7 respondents who had completed fewer than 50% of our questions, and we removed all entries of the 3 respondents who, by their Amazon Mturk ID, seemed to have participated in the study twice (resulting in the removal of 6 cases). Because we expected that reading the questions alone would take more than 2 minutes, we eliminated a total of 18 respondents who had spent 2 minutes or less on our survey, to guard against distortions from careless responding (e.g., Aruguete et al., 2019). Another 19 respondents missed our attention check (requiring respondents to select a particular response (“Please select ‘Somewhat agree.’”), and were subsequently eliminated. No fewer than 85 respondents had to be eliminated because they did not answer our open-ended question as to their current location in the U.S. (state), or they did provide nonsense answers, raising the concern that the entire response may have been generated by a bot (Stokel-Walker, 2018). We also eliminated 6 cases whose IP addresses appeared more than once in our data set.

**Sample Breakdown by State**

Our analysis sample included 633 respondents from California (*n* = 55), New York (*n* = 45), Illinois (*n* = 35), Texas (*n* = 34), Florida (*n* = 33), Nevada (*n* = 27), Georgia and Washington (each *n* = 21), Connecticut (*n* = 20), Maryland (*n* = 19), Colorado and North Carolina (each *n* = 18), Arizona and Ohio (each *n* = 16), New Jersey (*n* = 15), Michigan and Pennsylvania (each *n* = 14), Minnesota (*n* = 11), Alabama, Missouri, Utah, and Virginia (each *n* = 10), Louisiana, Montana, Nebraska, and Tennessee (each *n* = 9), Idaho, Kentucky and Maine (each *n* = 8), Arkansas, Iowa, Massachusetts, West Virginia and Wisconsin (each *n* = 7), and Hawaii, Indiana, Kansas, Mississippi, New Hampshire, New Mexico, Oklahoma, Oregon, Rhode Island, South Carolina and Wyoming (each *n* = 6).

**Representativeness of the sample**

By its nature, our sample was not a representative of the U.S. population, nor was there any deliberate effort to ensure that respondents from each state were a representative sample of the state’s population. By virtue of becoming an Mturk worker, respondents are part of a self-selected group. However, respondents from a particular state may still serve as representatives of that state, simply because there is no reason to assume any differential selection. In other words, even though Mturk workers might not be typical residents of their states, all respondents shared this characteristic, which was constant across states. This, in turn, allows Mturk workers to serve as representatives of their respective states (see Straus, 2009, for a general outline of this approach; see Saucier et al., 2018, for an approach involving Mturk).

**S2 Dependent variables**

Unless stated otherwise, respondents responded to all items on a five-point scale ranging from 1 *Strongly disagree* to 5 *Strongly agree*, with the midpoint 3 labeled *Neither agree nor disagree*.

**Mask wearing behavior**

*Past behavior*

“Thinking of the last 7 days, how often did you wear a mask when you left your house?”

*Behavioral Intent*

“Next time when I leave my house, I intend to wear a mask.”).

Respondents chose one of five responses: *Never*, *Sometimes*, *About half the time*, *Most of the time* and *Always*.

**Behavior change**

“I am willing to change my mask wearing behavior if people closest to me want me to.”

“I am willing to change my mask wearing behavior if people in my community want me to.”

These items were combined.

**Knowledge**

“How much do you feel you know about the novel coronavirus?”

Respondents described themselves on a 5-point scale ranging from 1 *Not knowledgeable at all* to 5 *Extremely knowledgeable*.

**Mask utility**

“It is in my own self-interest to wear a mask in public.”

“It is in my own interest if other people wear a mask in public.”

“It is in other people’s interests for me to wear a mask in public.”

“It is in my community’s interest if I wear a mask in public.”

“Wearing a mask protects me from becoming infected with coronavirus.”

“Wearing a mask protects others from becoming infected with coronavirus.”

“Wearing a mask protects the community from the coronavirus.”

These items were combined.

**Feeling of protection**

“Wearing a mask makes the people closest to me feel protected.”

“Wearing a mask makes others in my community feel protected.”

These items were combined.

**Social norms**

“Wearing a mask makes me feel proud.”

“Wearing a mask makes me feel respected.”

These items were combined.

**Social recognition**

*Trust in government*

“I do not trust the government officials who decide people should wear a mask or not in public.”

This item was reverse coded.

*Trust in public health officials*

“I have trust in the public health officials who are making the decision as to whether a mask is required or not.”

**Negative evaluation**

“I hate wearing a mask.”

**“**Wearing a mask is not necessary.”

These items were combined.

**Social image**

*Image of Strength*

“A mask is a sign of weakness.”

*In the eyes of non-benevolent others*

“I don’t want to give others the satisfaction of seeing me with a mask.”

**Low well-being**

“Wearing a mask makes me feel embarrassed.”

“Wearing a mask makes me feel depressed.”

“Wearing a mask makes me feel anxious.”

“Wearing a mask makes me feel isolated from other people.”

These items were combined.

**Freedom vs. civic duty**

“Having to wear a mask in public infringes on my freedom as a U.S. citizen.”

“By wearing a mask in public, I am doing my duty as a U.S. citizen.”

“I am philosophically opposed to wearing a mask.”

These items were combined.

**Voluntariness**

“In my community, people are not pressured to wear a mask; they make their own decision whether to wear a mask or not.”

**Self-Construal Items (sample items)**

***Independent Self-Construal***

“My personal identity independent of others, is very important to me.”

“I should be judged on my own merit.”

***Collective Interdependence Self-Construal***

“I will sacrifice my self-interest for the benefit of the group I am in.”

“Being accepted by other members of my group is important to me.”

***Relational Interdependence Self-Construal***

“When I feel very close to someone, it often feels to me like that person is an important part of who I am.”

“If a person hurts someone close to me, I feel personally hurt as well.”

Each of the three self-construal scales comprised five items, of which we provide two sample items. The complete scales can be found in Merhi (2021).

**S3 Cultural differences between U.S. states**

**Collectivism**

Vandello and Cohen (1999) generated their state-collectivism index based on eight different data sources: (a) the percentage of people living alone; (b) the ratio of divorce rate to marriage rate; (c) the percentage of elderly people living alone; (d) the percentage of people with no religious affiliation; (e) the average percentage of votes for the Libertarian party during the past four presidential elections; (f) the percentage of self-employed people, (g) the ratio of people carpooling to work compared to those driving alone; and (h) the percentage of households with grandchildren in them. For the first six variables, lower values indicate higher levels of collectivism. For the last two variables, higher valued indicate higher levels of state collectivism. The former six variables were reverse-scored to create the index.

Recent work by Kusano and Kemmelmeier (in press) revealed that, to the extent that data were available, between-state differences with regard to the eight components of Vandello and Cohen’s (1999) index were remarkably constant between the early 1990s and 2018.

**Tightness-Looseness**

Following the method originated by Vandello and Cohen (1999), Harrington and Gelfand (2014) generated and validated an index based on nine variables characterizing differences between states: (a) whether or not corporal punishment was legal in public schools; (b) the percentage of students who received corporal punishment in schools; (c) the date of executions (1976-2011); (d) the severity of punishment for violating drug laws pertaining to marijuana; (e) share of counties within each state that limited the sale of alcohol; (f) legality of same-sex civil unions; (g) percentage of state population that indicates that religion is an important part of their lives; (h) percentage of population without any religious affiliation; and (i) the percentage of the population who were not born in the U.S. (α = .84).

**S4 Supplementary Information pertaining to State-level Control Variables**

**State demographics.** Because age represents a critical risk factor for severe consequences of COVID-19, we also included the median age of each state as a predictor (see <https://worldpopulationreview.com/state-rankings/median-age-by-state>).

**Composition of the state legislators.** To account for latent political differences between states, we computed the share of state legislators (representatives and senators) who were members of the Democratic Party as of December 2020 (Ballotpedia, 2020). Because Nebraska has a non-partisan lower house, we only included their upper house in the proportion of Democratic Party members.

**COVID-19 threat.** Because there was considerable variance in the severity of the pandemic across U.S. states, we also included for each state the cumulative number of cases that had been reported to the CDC by July 20, 2020, when the present survey was launched. In a set of alternative analyses, we divided the state total by the state population to control for the share of the population that tested positive for SARS-CoV-2.

Table 2b revealed that U.S. honor states were significantly tighter than non-honor states. This is to be expected in light of the fact that the very concept of reputation presumes a shared basis of shared values and ideas as to what constitutes acceptable behavior. The correlation between honor and collectivism was only weak, with the association between tightness and collectivism being not much stronger. Remarkably, honor states and those high in tightness were markedly less affluent than those non-honor states and loose states.

**S5 Supplementary materials pertaining to Individual-level predictors**

African Americans scored slightly higher relative to Whites with regard to collective interdependence (see Coon & Kemmelmeier, 2001; Vargas & Kemmelmeier, 2013). Moreover, Asian Americans were slightly lower in terms of relational interdependence and conservatism than Whites. Notably, in terms of political orientation, our sample seemed to be middle of the road (*M* = 3.10 on a five-point scale) and unusually highly educated with 74% reporting having earned a college degree. Most were around 25-34 years old (46%).

**S6 Supplementary Information pertaining to Analytical Approach**

As our statistical model, we employed a two-level mixed effects model. The regression equations of the model were as follows:

Level 1

*Y*_ij_ = β_0j_ + β_1j_*X*_1ij_ + β_2j_*X*_2ij_ + β_3j_*X*_3ij_ + β_4j_*X*_4ij_ + β_mj_*C*_mij_ + e_ij_

where for every respondent *i* nested within state *j*, *X*_1_ is independence, *X*_2_ collective interdependence, *X*_3_ relational interdependence, and *X*_4_ represents political orientation (conservatism-liberalism). C represents a set of *m* individual-level control variables.

Level 2

β_0j_ = ɣ_00_ + ɣ_01_*Z*_1j_ + ɣ_00_*Z*_2j_ + ɣ_00_*Z*_3j_ + ɣ_01_*W*_nj_ + µ_0j_

where for every state j, Z_1_ is honor culture, Z_2_ tightness-looseness, Z_3_ collectivism, and *W_n_* represents a set of *n* state-level control variables.

**S7 Supplementary Analyses pertaining to Perceived Utility**

We conducted analyses in which we treated each of the seven items of our perceived utility index as separate dependent variables. Notwithstanding that the items loaded on a singular factor, the content of the item refer to different refers to different types of interests, namely, self-interest and other-interest (see Table S1 & S2 below).

**Table S7.1**

Perceived Utility Facets

_____________________________________________________________________________________

Mask Mask Mask

Protects Me Protects Others Protects Community

______________ ______________ ______________

*b* (*se*) *b* (*se*) *b* (*se*)

_____________________________________________________________________________________

Intercept 3.88^***^ (*0.24*) 4.48^***^ (*0.19*) 4.39^***^ (*0.19*)

*Individual-Level*

Female (Male = 0) -0.11 (*0.09*) -0.14^+^ (*0.07*) -0.06 (*0.07*)

Education (High School = 0)

Some college 0.16 (*0.18*) -0.04 (*0.14*) -0.05 (*0.14*)

College 0.15 (*0.16*) -0.05 (*0.13*) -0.07 (*0.13*)

Advanced Deg. 0.09 (*0.19*) -0.12 (*0.15*) -0.06 (*0.15*)

Age (18-24)

25-34 -0.01 (*0.18*) -0.01 (*0.14*) -0.03 (*0.14*)

35-44 -0.10 (*0.19*) -0.08 (*0.15*) -0.09 (*0.15*) 45-54 -0.14 (*0.20*) -0.11 (*0.16*) -0.15 (*0.16*) 55-64 -0.01 (*0.24*) -0.19 (*0.19*) -0.26 (*0.19*) 65-74 -0.37 (*0.36*) -0.16 (*0.29*) -0.56^+^ (*0.29*)

Race/Ethnicity (White = 0)

Asian 0.02 (*0.19*) -0.03 (*0.16*) -0.04 (*0.16*)

Black 0.30^*^ (*0.13*) -0.16 (*0.11*) 0.10 (*0.11*)

Latinx -0.04 (*0.21*) -0.13 (*0.16*) 0.04 (*0.16*) Other -0.05 (*0.47*) 0.41 (*0.37*) 0.40 (*0.38*)

Independence -0.06 (*0.08*) **0.17^**^ (*0.06*)** **0.18^***^ (*0.06*)**

Collective interdep. **0.39^***^ (*0.08*)** **0.36^***^ (*0.06*) 0.47^***^ (*0.06*)**

Relational interdep. 0.12 (*0.07*) **0.13**^*^ (*0.06*) 0.06 (*0.06*)

Conservatism **-0.13^***^ (*0.03*) -0.24^***^ (*0.03*)** -**0.26^***^ (*0.03***)

Mask Mandatory 0.07 (*0.11*) 0.04 (*0.09*) 0.05 (*0.09*)

*State-Level*

Honor State (No = 0) -0.043 (*0.110*) -0.090 (*0.084*) -0.005 (*0.077*)

Tightness 0.006 (*0.005*) 0.008^+^ (*0.004*) 0.001 (*0.004*)

Collectivism 0.009^+^ (*0.005*) 0.007^+^ (*0.004*) 0.005 (*0.004*)

GSP -0.004 (*0.006*) 0.002 (*0.005*) -0.002 (*0.004*)

Inequality 0.005 (*0.003*) -0.003 (*0.020*) 0.029 (*0.021*)

*Variance Components*

State 0.03 0.01 0.00

Residual 1.02 0.66 0.67

*Model Fit*

AIC 1847.53 1593.34 1590.95

BIC 1962.07 1707.89 1705.48

-2 Log Likelihood 1795.53 1541.34 1538.95

Marginal R^2^ 0.15 0.24 0.27

Conditional R^2^ 0.16 0.25 0.27

*N* 605 605 605

_______________________________________________________________________________________

^+^*p* < .10, **p* < .05; ***p* < .01; ****p* < .001.

*Note*: For our main predictor variables, coefficients and their standard errors are bolded, if they are significant at a minimum of *p* < 0.05.

**Table S7.2**

Perceived Utility Facets

_____________________________________________________________________________________

Own Self Interest In other’s Interest Own interest Community’s interest

To Wear Mask To Wear Mask For Other wear mask To Wear Mask

_______________ _______________ _______________ _______________

*b* (*se*) *b* (*se*) *b* (*se*) *b* (*se*)

_____________________________________________________________________________________

Intercept 4.25^***^ (*0.22*) 4.61^***^ (*0.19*) 4.45^***^ (*0.22*) 4.62^***^ (*0.19*)

*Individual-Level*

Female (Male = 0) -0.14 (*0.08*) -0.08 (*0.07*) -0.09 (*0.08*) -0.09 (*0.07*)

Education (High School = 0)

Some college -0.03 (*0.16*) -0.09 (*0.14*) -0.04 (*0.16*) -0.07 (*0.14*)

College 0.01 (*0.15*) -0.09 (*0.13*) -0.07 (*0.15*) -0.09 (*0.13*)

Advanced Deg. 0.03 (*0.17*) -0.09 (*0.15*) 0.03 (*0.17*) -0.07 (*0.15*)

Age (18-24)

25-34 0.08 (*0.16*) 0.04 (*0.14*) 0.01 (*0.16*) -0.03 (*0.14*)

35-44 0.04 (*0.17*) 0.03 (*0.14*) -0.10 (*0.17*) -0.05 (*0.15*)

45-54 -0.06 (*0.19*) 0.02 (*0.16*) -0.08 (*0.18*) -0.03 (*0.16*)

55-64 -0.09 (*0.21*) -0.19 (*0.18*) -0.12 (*0.21*) <-0.01 (*0.19*)

65-74 0.10 (*0.32*) -0.04 (*0.28*) -0.20 (*0.32*) -0.09 (*0.28*)

Race/Ethnicity (White = 0)

Asian -0.14^+^ (*0.08*) -0.07 (*0.15*) -0.30 (*0.18*) -0.03 (*0.15*)

Black 0.10 (*0.18*) -0.03 (*0.11*) 0.12 (*0.12*) 0.03 (*0.11*)

Latinx 0.24^+^ (*0.12*) 0.14 (*0.16*) 0.04 (*0.19*) -0.08 (*0.16*)

Other 0.30 (*0.42*) 0.45 (*0.36*) 0.52 (*0.42*) 0.43 (*0.37*)

Independence **0.23^**^ (*0.07*)** **0.22^***^ (*0.06*)** **0.25^***^ (*0.07*)** **0.26^***^ (*0.06*)**

Collective interdep. **0.40^***^ (*0.07*)** **0.30^***^ (*0.06*) 0.40^***^ (*0.07*)** **0.31^***^ (*0.06*)**

Relational interdep. 0.05 (*0.07*) 0.05 (*0.06*) 0.07 (*0.07*) 0.09 (*0.06*)

Conservatism **-0.19^***^ (*0.03*) -0.23^***^ (*0.03*)** -**0.25^***^ (*0.03***) -**0.23^***^ (*0.03*)**

Mask Mandatory 0.10 (*0.10*) -0.05 (*0.09*) 0.04 (*0.10*) -0.04 (*0.09*)

*State-Level*

Honor State (No = 0) -0.087 (*0.092*) **-0.167^*^ (*0.075*)** -0.074 (*0.111*) **-0.168^*^ (*0.076*)**

Tightness 0.002 (*0.004*) **0.007^*^ (*0.003*)** <0.001 (*0.005*) 0.007^+^ (*0.003*)

Collectivism 0.003 (*0.005*) **0.008^*^ (*0.004*)** 0.010^+^ (*0.005*) 0.007^+^ (*0.004*)

GSP -0.002 (*0.005*) -0.003 (*0.004*) -0.005 (*0.006*) -0.002 (*0.004*)

Inequality 0.023 (*0.026*) -0.011 (*0.021*) <-0.001 (*0.030*) 0.003 (*0.020*)

*Variance Components*

State 0.01 0.00 0.03 <0.01

Residual 0.85 0.62 0.82 0.65

*Model Fit*

AIC 1736.55 1551.64 1728.88 1579.52

BIC 1851.08 1666.14 1843.41 1694.06

-2 Log Likelihood 1682.55 1499.64 1676.88 1527.52

Marginal R^2^ 0.18 0.22 0.21 0.23

Conditional R^2^ 0.18 0.22 0.24 0.23

*N* 605 604 605 605

_______________________________________________________________________________________

^+^*p* < .10, **p* < .05; ***p* < .01; ****p* < .001.

*Note*: For our main predictor variables, coefficients and their standard errors are bolded, if they are significant at a minimum of *p* < 0.05.

**S8 Individual-level mediation analyses**

Though we provide specific mediation hypotheses, recall that any reliable mediation link merely demonstrates that our data are consistent with the notion that a particular variable functions as a causal mediator; yet, indirect effects computed in the context of cross-sectional survey data can never serve as definitive evidence of a causal relationship (Fiedler et al., 2012).

Based on Monte Carlo simulations carried out based on Schoemann et al. (2017), we sought to determined levels of effective power for all indirect effects summarized in Figure 1. Note that we did not have a way to generate such Monte Carol simulations for our multilevel data. By resorting to Schoemann et al. (2017) for our determination of power we effectively ignored our multilevel design. However, because our ICC was small (maximum .05), with regard to level-1 analyses the difference between ordinary-least square regression and multilevel regression must be small. Moreover, because Schoemann et al.’s approach (2017) does allow for no more than two simult­aneous mediators, we computed power for each individual indirect effect, as was the case for the statistical effects of conservatism onto behavior change (Figure 1c). Overall, our power estimates are only approximations, with the true achieved power likely being slightly lower. This is of minor concern as long as power estimates remains comfortably above the conventional threshold of .80.

Concerning the indirect effects depicted in Figure 1a, our Monte Carlo simulations showed the power for the test of each indirect effects to be approaching β = 1.00. The only exception was the indirect effect of independence through perceived utility onto past behavior. For this particular indirect effect test we only had a power of β = .96. Similar result emerged for Figure 1b: all tests of indirect effects approached β = 1.00, though for the test involving independence, achieved power was β = .95. With regard to the indirect effects depicted in Figure 1c, four of five tests of indirect effects in which conservatism served as predictor had a power approaching β = 1.00. The fifth test of the indirect effect through perceived utility only achieved a power of β = .94. The test of the indirect effect involving collective interdependence achieve a power of β = .97. Surprisingly, though the indirect effect involving independence had emerged as statistically significant, according to our Monte Carlo simulations the power achieved in this test was only minimal, β = .05.

**S9 State-level mediation analyses**

Note that this mediation analysis was underpowered. Based on Monte Carlo simulations carried out based on Schoemann et al. (2017), at *n* = 45, the test of an indirect effect of collectivism on past behavior (aggregated) through perceived utility (aggregated) was adequately powered, even if the power was lower than desirable, β = .73. The test of an indirect effect through social norms (aggregated) turned out to be uninterpretable, β = .09; not surprisingly, this indirect effect did not emerge as statistically significant.

References

Aruguete, M. S., Huynh, H., Browne, B. L., Jurs, B., Flint, E., & McCutcheon, L. E. (2019). How serious is the ‘carelessness’ problem on Mechanical Turk?. *International Journal of Social Research Methodology*, *22*(5), 441-449.

Bai, H. (2018). Evidence that a large amount of low quality responses on MTurk can be detected with repeated GPS coordinates. Retrieved December 27, 2020, from Sights + Sounds website: <http://www.maxhuibai.com/1/post/2018/08/evidence-that-responses-from-repeating-gps-are-random.html>

Ballotpedia, (2020). Election results, 2020: “State legislative chambers that changed party control.” Retrieved from [https://ballotpedia.org/Partisan_composition_of_state_legislatures](https://nam04.safelinks.protection.outlook.com/?url=https%3A%2F%2Fballotpedia.org%2FPartisan_composition_of_state_legislatures&data=04%7C01%7Cmarkusk%40unr.edu%7Ca23a5909e0674b2904b808d8ad57f715%7C523b4bfc0ebd4c03b2b96f6a17fd31d8%7C1%7C0%7C637449940622374875%7CUnknown%7CTWFpbGZsb3d8eyJWIjoiMC4wLjAwMDAiLCJQIjoiV2luMzIiLCJBTiI6Ik1haWwiLCJXVCI6Mn0%3D%7C1000&sdata=gKWFJDZ9MhCDPQsy6UpBVzHg%2B0VFRkfqzEX12epkeCc%3D&reserved=0)

Coon, H. M., & Kemmelmeier, M. (2001). Cultural orientations in the United States: (Re-) examining differences among ethnic groups. *Journal of Cross-Cultural Psychology, 32*, 348-364.

Chmielewski, M., & Kucker, S. C. (2020). An MTurk crisis? Shifts in data quality and the impact on study results. *Social Psychological and Personality Science*, *11*(4), 464-473.

Fiedler, K., Schott, M., & Meiser, T. (2011). What mediation analysis can (not) do. *Journal of Experimental Social Psychology*, *47*(6), 1231-1236.

Harrington, J. R., & Gelfand, M. J. (2014). Tightness–looseness across the 50 United States. *Proceedings of the National Academy of Sciences, 111(22)*, 7990-7995.

Kennedy, R., Clifford, S., Burleigh, T., Waggoner, P. D., Jewell, R., & Winter, N. J. (2020). The shape of and solutions to the MTurk quality crisis. *Political Science Research and Methods*, *8*(4), 614-629.

Kusano, K., & Kemmelmeier, M. (in press). Cultural change through niche construction: A multilevel approach to investigate the interplay between cultural change and infectious disease. *American Psychologist*.

Merhi, H. (2021). The effect of self-construal on the level of engagement with cause-related marketing campaigns. [Unpublished doctoral dissertation]. Barcelona: Universitat Autònoma de Barcelona.

Saucier, D. A., Miller, S. S., Martens, A. L., O'Dea, C. J., & Jones, T. L. (2018). Individual differences explain regional differences in honor-related outcomes. *Personality and Individual Differences*, *124*, 91-97.

Scherbaum, C. A., & Ferreter, J. M. (2009). Estimating statistical power and required sample sizes for organizational research using multilevel modeling. *Organizational Research Methods*, *12*(2), 347-367.

Schoemann, A. M., Boulton, A. J., & Short, S. D. (2017). Determining power and sample size for simple and complex mediation models. *Social Psychological and Personality Science*, *8*(4), 379-386.

Snijders, T. A. B. (2005). Power and sample size in multilevel linear models. In B. S. Everitt & D. C. Howell (Eds.), *Encyclopedia of statistics in behavioral science* (Vol. 3, pp. 1570–1573). Wiley.

Straus, M. A. (2009). The national context effect: An empirical test of the validity of cross-national research using unrepresentative samples. *Cross-Cultural Research*, *43*(3), 183-205.

Stokel-Walker, C. (2018, October 1). Bots on Amazon’s Mechanical Turk are ruining psychology studies. Retrieved December 27, 2020, from New Scientist website: <https://www.newscientist.com/article/2176436-bots-on-amazons-mechanical-turk-are-ruining-psychology-studies/>

Vandello, J. A., & Cohen, D. (1999). Patterns of individualism and collectivism across the United States. *Journal of Personality and Social Psychology, 77*, 279-292.

Vargas, J. H., & Kemmelmeier, M. (2013). Ethnicity and contemporary American culture: A meta-analytic investigation of horizontal–vertical individualism–collectivism. *Journal of Cross-Cultural Psychology*, *44*, 195-222. DOI: 10.1177/0022022112443733

1. This approach may have resulted in the elimination of U.S. residents using a virtual private network (VPN), who only appeared to be located outside of the U.S. However, because it was impossible to distinguish such respondents from those physically located outside of the U.S. based on the IP address, the decision was made to err on the side of caution. [↑](#footnote-ref-1)
